# The PRolaCT studies — a study protocol for a combined randomised clinical trial and observational cohort study design in prolactinoma

**DOI:** 10.1186/s13063-021-05604-y

**Published:** 2021-09-25

**Authors:** Ingrid M. Zandbergen, Amir H. Zamanipoor Najafabadi, Iris C. M. Pelsma, M. Elske van den Akker-van Marle, Peter H. L. T. Bisschop, H. D. Jeroen Boogaarts, Arianne C. van Bon, Bakhtyar Burhani, Saskia le Cessie, Olaf M. Dekkers, Madeleine L. Drent, Richard A. Feelders, Johan P. de Graaf, J. Hoogmoed, Kitty K. Kapiteijn, Melanie M. van der Klauw, Willy-Anne C. M. Nieuwlaat, Alberto M. Pereira, Aline M. E. Stades, Annenienke C. van de Ven, Iris M. M. J. Wakelkamp, Wouter R. van Furth, Nienke R. Biermasz, on behalf of the Dutch Prolactinoma Study Group

**Affiliations:** 1 Department of Neurosurgery, Leiden University Medical Center, Albinusdreef 2, 2333ZA Leiden, The Netherlands; 2 Department of Medicine, Division of Endocrinology, Leiden University Medical Center, Albinusdreef 2, 2333ZA Leiden, The Netherlands; 3 Department of Medicine, Center for Endocrine Tumours Leiden, Leiden University Medical Center, Albinusdreef 2, 2333ZA Leiden, The Netherlands; 4 Department of Biomedical Data Sciences, Section Medical Decision Making, Leiden University Medical Center, Albinusdreef 2, 2333ZA Leiden, The Netherlands; 5 Department of Medicine, Division of Endocrinology, Amsterdam University Medical Center - location AMC, Meibergdreef 9, 1105AZ Amsterdam, The Netherlands; 6 Department of Neurosurgery, Radboud University Medical Center, Geert Grooteplein Zuid 10, 6525GA Nijmegen, The Netherlands; 7 Department of Internal Medicine, Rijnstate, Wagnerlaan 55, 6815AD Arnhem, The Netherlands; 8 Department of Neurosurgery, Elisabeth-Tweesteden Ziekenhuis, Hilvarenbeekseweg 60, 5022GC Tilburg, The Netherlands; 9 Department of Clinical Epidemiology, Leiden University Medical Center, Albinusdreef 2, 2333ZA Leiden, The Netherlands; 10 Department of Medicine, Division of Endocrinology, Amsterdam University Medical Center - location VUmc, De Boelelaan 1118, 1081HZ Amsterdam, The Netherlands; 11 Department of Medicine, Division of Endocrinology, Erasmus Medical Center, Doctor Molewaterplein 40, 3015GD Rotterdam, The Netherlands; 12 Dutch Pituitary Foundation, PO BOX 1014, 3860BA Nijkerk, The Netherlands; 13 Department of Neurosurgery, Amsterdam University Medical Center - location AMC, Meibergdreef 9, 1105AZ Amsterdam, The Netherlands; 14 Department of Gynaecology, Reinier de Graaf Gasthuis, Reinier de Graafweg 5, 2625AD Delft, The Netherlands; 15 Department of Medicine, Division of Endocrinology, University Medical Center Groningen, Hanzeplein 1, 9713GZ Groningen, The Netherlands; 16 Department of Internal Medicine, Elisabeth-Tweesteden Ziekenhuis, Hilvarenbeekseweg 60, 5022 GC Tilburg, The Netherlands; 17 Department of Medicine, Division of Endocrinology, University Medical Center Utrecht, Heidelberglaan 100, 3584CX Utrecht, The Netherlands; 18 Department of Medicine, Division of Endocrinology, Radboud University Medical Center, Geert Grooteplein Zuid 10, 6525GA Nijmegen, The Netherlands; 19 Department of Internal Medicine, St. Antonius Ziekenhuis, Koekoekslaan 1, 3435CM, Nieuwegein, The Netherlands

**Keywords:** Prolactinoma, Pituitary tumour, Dopamine agonist, Endoscopic transsphenoidal resection, Randomised clinical trial, Observational cohort

## Abstract

**Background:**

First-line treatment for prolactinomas is a medical treatment with dopamine agonists (DAs), which effectively control hyperprolactinaemia in most patients, although post-withdrawal remission rates are approximately 34%. Therefore, many patients require prolonged DA treatment, while side effects negatively impact health-related quality of life (HRQoL). Endoscopic transsphenoidal resection is reserved for patients with severe side effects, or with DA-resistant prolactinoma. Surgery has a good safety profile and high probability of remission and may thus deserve a more prominent place in prolactinoma treatment. The hypothesis for this study is that early or upfront surgical resection is superior to DA treatment both in terms of HRQoL and remission rate in patients with a non-invasive prolactinoma of limited size.

**Methods:**

We present a combined randomised clinical trial and observational cohort study design, which comprises three unblinded randomised controlled trials (RCTs; PRolaCT-1, PRolaCT-2, PRolaCT-3), and an observational study arm (PRolaCT-O) that compare neurosurgical counselling, and potential subsequent endoscopic transsphenoidal adenoma resection, with current standard care. Patients with a non-invasive prolactinoma (< 25 mm) will be eligible for one of three RCTs based on the duration of pre-treatment with DAs: PRolaCT-1: newly diagnosed, treatment-naïve patients; PRolaCT-2: patients with limited duration of DA treatment (4–6 months); and PRolaCT-3: patients with persisting prolactinoma after DA treatment for > 2 years. PRolaCT-O will include patients who decline randomisation, due to e.g. a clear treatment preference. Primary outcomes are disease remission after 36 months and HRQoL after 12 months.

**Discussion:**

Early or upfront surgical resection for patients with a limited-sized prolactinoma may be a reasonable alternative to the current standard practice of DA treatment, which we will investigate in three RCTs and an observational cohort study. Within the three RCTs, patients will be randomised between neurosurgical counselling and standard care. The observational study arm will recruit patients who refuse randomisation and have a pronounced treatment preference. PRolaCT will collect randomised and observational data, which may facilitate a more individually tailored practice of evidence-based medicine.

**Trial registration:**

US National Library of Medicine registry (ClinicalTrials.gov) NCT04107480. Registered on 27 September 2019, registered retrospectively (by 2 months).

**Supplementary Information:**

The online version contains supplementary material available at 10.1186/s13063-021-05604-y.

## Administrative information

Trial registration

Primary identifying number:

CCMO-Registry ID: NL63919.058.18

Secondary identifying numbers:

ZonMw Grant ID: 843002806

ClinicalTrials.gov ID: NCT04107480

Protocol version: 5

Issue date: 7 Jan 2021

Protocol amendment number: 3

Trials Sponsor (coordinating centre): Leiden University Medical Center

## Background

Prolactin-producing pituitary adenomas typically cause functional hyperprolactinaemia, which is characterised by galactorrhoea and hypogonadism, and may therefore result in subfertility [1]. Additionally, non-specific symptoms, such as fatigue, and psychological and neurocognitive complaints have been described [1]. Although a rare condition, prolactinomas account for over half (32–66%) of all pituitary adenomas [1]. Prolactinomas are found 3 times more frequent in women than in men with a peak incidence between the age of 25 and 35 years [2, 3]. The majority of prolactinomas are subcategorised, based on the size on magnetic resonance imaging (MRI), as microprolactinomas (< 10 mm; 80%), but they may also present as macro (10–40 mm) or giant (> 40 mm) and invasive tumours, which may compromise visual function due to compression of the optic chiasm, nerves, or tracts [2].

Prolactinoma treatment is primarily targeted at reduction of symptoms and restoration of gonadal status and fertility by normalising prolactin levels [4]. As it is a highly effective and non-invasive treatment option, medical treatment with a dopamine agonist (DA) is recommended as first-line treatment for almost all prolactinoma patients, while endoscopic transsphenoidal resection is reserved for those patients with (severe) DA intolerance or in case of DA-resistant prolactinoma [4].

Although DA treatment results in normalisation of prolactin levels, and subsequent reduction of symptoms in 81% of patients, the 2-year remission rate after DA withdrawal remains low (34%) and, therefore, long-term medical treatment should be expected [5]. DA treatment is usually well tolerated, although orthostatic hypotension and gastrointestinal complaints are frequently reported as side effects. DAs are furthermore associated with psychological side effects, which are mild in 40%, but more severe in up to 5–10% of patients [5–13]. Particularly, impulse control disorders (ICDs), e.g. pathologic gambling, are acknowledged as a typical side effect [13–16]. The heterogenous pattern of side effects and prolactinoma symptomatology may (in part) explain that both untreated (symptomatic) and medically treated prolactinoma patients have reported a decreased physical and mental health-related quality of life (HRQoL) [5, 17–22].

Although clearly more invasive, endoscopic pituitary surgery has a well-established good safety profile for prolactinomas of limited size. Long-term morbidity from complications occurs in less than 2–3% of patients (mostly due to hypopituitarism or permanent diabetes insipidus), whereas transient perioperative complications, such as cerebrospinal fluid leakage and transient diabetes insipidus, occur in less than 5 and 15% of patients, when surgery is performed by experienced neurosurgeons [5, 17, 23–27]. Resulting in immediate cure in 80 to 90% of prolactinoma patients, endoscopic transsphenoidal surgery is also a highly effective treatment [5, 17, 23–25]. Moreover, the vast majority of postoperative patients will not need long-term DA treatment, since recurrence rates after surgery are 4–15% [5, 17, 28]. Interestingly, two recent simulation studies have estimated that endoscopic surgery could be a more cost-effective treatment when compared to cabergoline, the most effective DA [29, 30].

With improvement of the endoscopic transsphenoidal surgical technique, increasing evidence of potentially detrimental side effects of DAs, and disappointing remission rates after a period of at least 2 years of DA treatment, equipoise may exist for these treatment options, particularly for patients with the greatest likelihood for successful complete adenoma resection (i.e. clearly visible, of limited size, and non-invasive tumours). Nevertheless, the efficacy-to-safety ratios of surgery and medical treatment are highly different and incomparable, so HRQoL after surgery (without DA in most cases) and risk of complications should be weighed against HRQoL during (often long-term) medical treatment and its potential side effects. However, there are currently only a handful of retrospective observational studies published on this topic with major drawbacks, such as selection bias and insufficient and inappropriate correction for confounding variables [5]. Therefore, we believe that this is the right moment in time to perform a randomised controlled trial (RCT) comparing both treatment strategies.

We hypothesise that endoscopic transsphenoidal adenoma resection as a first-line, or equally valid second-line, treatment is superior to standard care (DA treatment) for patients with a visible prolactinoma of limited size (< 25 mm, no tissue invasion). The primary outcome parameters are (1) HRQoL measured after 12 months of follow-up (i.e. 12 months after randomisation or, in PRolaCT-O, study baseline) and (2) the proportion of patients in remission at 36 months of follow-up/study baseline.

## Methods

### Study design

This combined randomised clinical trial and observational cohort study design comprises three unblinded parallel superiority prolactinoma RCTs (PRolaCT-1, PRolaCT-2, PRolaCT-3) and an observational study arm (PRolaCT-O) that will compare counselling for endoscopic transsphenoidal adenoma resection to standard care (DA treatment) in patients with a prolactinoma of limited size (i.e. < 25 mm, no tissue invasion):

1. PRolaCT-1 compares counselling for surgical resection as a first-line treatment in newly diagnosed, treatment-naïve prolactinoma patients.
2. PRolaCT-2 compares counselling for surgical resection as an early second-line treatment in patients who have had DA treatment for a limited time period (4–6 months).
3. PRolaCT-3 compares counselling for surgical resection as an equal second-line treatment in patients who have persisting prolactinoma, i.e. recurring hyperprolactinaemia and persisting pituitary adenoma on MRI, after DA treatment for a long period of time (> 2 years).
4. PRolaCT-O evaluates counselling for surgical resection as an equally valid second-line treatment and standard care in an observational study setting, aiming to include all patients eligible for PRolaCT-1, PRolaCT-2, and PRolaCT-3 who do not participate in the RCTs because of a strong patient and/or physician treatment preference, and/or because the patient does not consent to randomisation.

All interventions and study procedures are otherwise identical for the three RCTs and the observational cohort.

Since some patients will be unwilling to be randomised for various reasons, such as a strong preference for either surgery or DA treatment, there is a risk that for the RCT a selected subpopulation is recruited, excluding patients with more pronounced side effects of DA treatment or those with an adenoma that is expected to be relatively difficult to resect completely. Especially outcomes in HRQoL might be attenuated or overestimated and there is a risk that the results cannot be generalised to all prolactinoma patients. The PRolaCT-O observational arm was added to provide a fall-back mechanism for evaluation of primary and secondary study objectives in case recruitment would prove to be challenging and to provide data for the evaluation of a possible selection bias in the RCTs, and is designed to collect real-life data about the eligible cohort. The combined RCT and observational cohort approach was received well by our advisory board (including a patient representative) and better reflects clinical decision-making in real life, as the MDT or treating physician may also have a preference due to tumour- and patient-specific characteristics. Furthermore, the prospective data collection of PRolaCT-O includes comprehensive information about expected confounders, such as duration and effectiveness of prior DA treatment, the amount and severity of side effects of DA treatment, and expected difficulty of surgery as judged by two blinded neurosurgeons.

#### Study setting

Endoscopic surgery is centralised in a few tertiary expert neurosurgical centres, while endocrine care for prolactinoma is delivered by endocrinologists and gynaecologists in all hospitals. For an expected enrolment period of 3 years, starting June 2019, recruitment for PRolaCT operates from five participating neurosurgical reference centres and uses regional multidisciplinary networks to recruit patients from the referring hospitals. PRolaCT is furthermore intended to expand to other reference centres in the Netherlands and internationally to the UK, France, and Germany, in collaboration with European Networks.

### Study population

Eligibility to participate with one of the RCTs or PRolaCT-O is based on the following inclusion and exclusion criteria.

#### Inclusion criteria

- At least 18 years of age
- A history of signs and symptoms matching with prolactinoma
- Hyperprolactinaemia, defined as a prolactin level ≥2 times the local laboratory maximum, present at the time of enrolment (PRolaCT-1) or present < 12 months before enrolment (PRolaCT-2 and PRolaCT-3)
- No clear alternative explanation for hyperprolactinaemia, e.g. medication use
- Presence of a clearly identifiable pituitary mass on MRI not invading the cavernous sinus and with a maximum diameter ≤25 mm. A representative MRI at the time of randomisation is required. This MRI should generally not be older than 12 months in PRolaCT-3 and 2 months in PRolaCT-1 and PRolaCT-2
- Defining inclusion criteria for the three RCTs are:
  - PRolaCT-1: no prior prolactinoma treatment
  - PRolaCT-2: prior DA treatment for 4–6 months or
  - PRolaCT-3: prior DA treatment for at least 2 years and having conducted at least one withdrawal attempt within 12 months prior to study recruitment (if the withdrawal attempt took place > 6 months before study inclusion, another withdrawal attempt is performed before randomisation to confirm prolactinoma persistence)

#### Exclusion criteria

- Contraindication for one of the treatment modalities, e.g. severe side effects of DA treatment or DA-resistant prolactinoma
- Contraindications to surgery, or a clear indication for surgical resection
- Pregnancy at the time of randomisation
- Clinical diagnosis of acromegaly
- Prior radiotherapy of the pituitary gland area
- Severe renal failure (eGFR < 30 ml/min)
- Insufficient understanding of the Dutch and English language

**Fig. 1:**
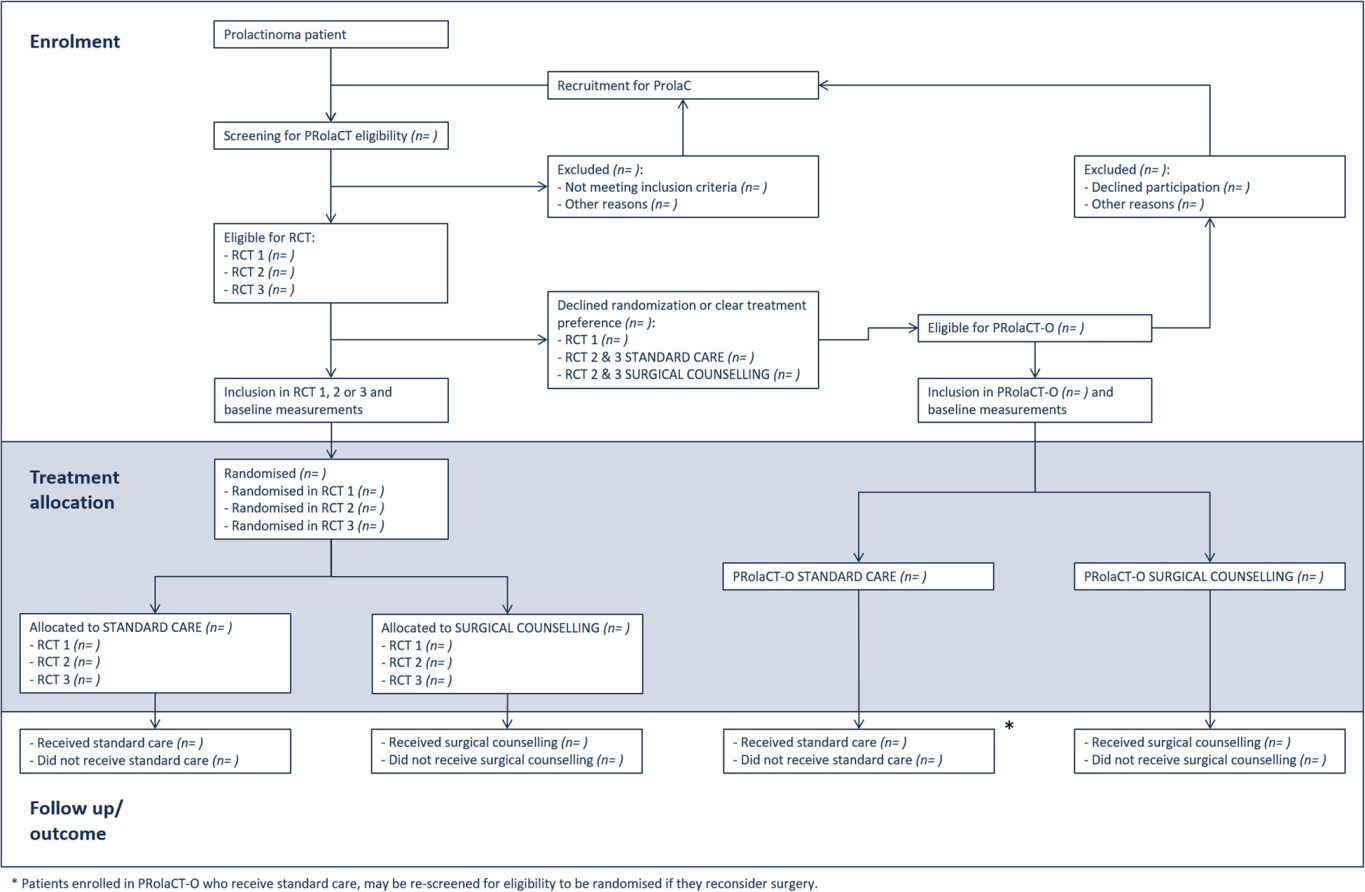
Overview of patient recruitment and treatment allocation

Patients eligible for participation in one of the RCTs, but do not consent to randomisation because there is a clear patient preference for either DA treatment or surgery, are considered for participation in PRolaCT-O, also see Fig. 1.

All prolactinoma patients are screened for eligibility to participate with one of the RCTs. Patients who decline randomisation or have a strong treatment preference are approached for PRolaCT-O. All ineligible patients will be approached for ProlaC, an additional registry including all prolactinoma patients (e.g. invasive tumours, pregnant patients).

### Patient identification and enrolment

Eligible patients are discussed within a regional multidisciplinary network, operating from the participating neurosurgical expertise centres. RCT eligibility is confirmed by the regional MDT. The counselling of new, eligible patients will be performed in the following order: first, willingness to participate in the RCT (PRolaCT-1, 2, 3) will be assessed, followed by willingness to be included in the intensive observational cohort with shared decision-making (PRolaCT-O). Patients who fulfil RCT eligibility but do not consent to randomisation or have a clear preference for standard care (PRolaCT-1) or either treatment modality (PRolaCT-2 and PRolaCT-3) are approached for participation in the observational arm, PRolaCT-O, which is depicted in Fig. 1. Patients who were approached for PRolaCT-1, but do not participate with PRolaCT, may be approached again for participation in PRolaCT-2 or PRolaCT-3, when the duration of DA treatment fulfils the inclusion criteria of the respective RCT. Likewise, patients who are enrolled in PRolaCT-O and receive standard care may be approached for participation in PRolaCT-2 or PRolaCT-3 if they reconsider surgical treatment. Written informed consent will be obtained from all participants prior to study inclusion.

### Treatment allocation

Within each RCT (PRolaCT-1, PRolaCT-2, or PRolaCT-3), participants are randomly allocated in a 1:1 ratio to either neurosurgical counselling (with potential subsequent surgery when desired) or standard care with DA. Randomisation is performed in blocks of sizes varying between 4, 6, and 8 patients and is stratified by sex to equally divide male participants and by tumour size (< 10 mm versus ≥ 10 mm). Randomisation is performed centrally in the LUMC using the built-in randomisation tool of the web-based database programme (Castor Electronic Data Capture (EDC), Castor, Amsterdam, the Netherlands). Investigators, treating physicians, and participants will not be blinded to the outcomes of randomization. With the use of varying block sizes, the allocation sequence is kept concealed by the randomisation programme. In PRolaCT-O, treatment choice, based on patient and physician preference, is solely recorded and may be DA treatment as a first- or second-line treatment, or surgical counselling as a second-line treatment (thus not in newly diagnosed patients). For all studies, the interpreters of MRIs, ophthalmologic assessments, and surgical difficulty will be blinded when possible. Unblinding will not occur.

### Treatment

#### Intervention: surgical counselling

Participants who are randomised for the intervention group will receive extensive and personalised neurosurgical counselling by a neurosurgeon and an endocrinologist with specific expertise in pituitary care (preferably a combined consultation). All potential benefits and risks of surgery are discussed in a semi-standardised manner, offering both standardised information on surgical intervention and detailed expert opinion regarding the patient’s specific situation. This consultation may theoretically result in advising the patient to refrain from surgery during the shared decision-making process, despite the patient’s previous eligibility “on paper”. Moreover, based on information gathered during the counselling, the MDT may advise against surgery and propose standard treatment as a better alternative in this individualised approach. After counselling, the patient is asked for consent for surgery as is a standard preoperative requirement regardless of study participation. Participants in the intervention group may thus decide not to proceed with the surgical intervention. Participants who do not consent to surgery will continue the RCT in the intervention group, but receive treatment according to standard care. Patients receiving standard of care may be referred back to their own physician following the counselling.

When a participant and the MDT agree to surgery, an endoscopic transsphenoidal surgical resection of the prolactinoma is performed according to standard practice in an expertise centre. Patients are hospitalised for 2–5 days postoperatively. In the first weeks after discharge from the hospital, frequent contact by phone or visits to the outpatient clinic are needed to check for complications and recovery of the patient, according to the standards of care of the neurosurgical centre. The frequency of these visits is gradually reduced to (half-)yearly visits depending on the patient’s well-being.

#### Control: standard care

All participants who are randomised for standard care will receive treatment as usual from their own endocrinologist or gynaecologist as described by the US Endocrine Society [4].

First-line treatment is with DA, of which cabergoline is used most often; alternatives are bromocriptine and quinagolide. The dosage is usually titrated to achieve a normal or suppressed prolactin level in combination with restoration of the gonadal axis. DA treatment is discontinued after 2 years of treatment, i.e. 24 months after randomisation, unless a normal prolactin level cannot be achieved, and is restarted when prolactin levels rise after the medication is discontinued. As is customary in standard care, patients in the control group may be offered surgery in case of DA intolerance (side effects) or an insufficient response to DA treatment.

All participants may use any form of co-medication when medically indicated. Surgery is not performed on a pregnant participant, unless specifically indicated. Women who are pregnant at the time of randomisation are therefore not eligible for inclusion in an RCT. If a patient enrolled in one of the RCTs becomes pregnant after randomisation, she will remain in the trial, but treatment will be adapted, according to standard clinical care.

Our intervention protocol has been conceived in agreement with current standards of care for prolactinoma patients with an indication for surgery. Therefore, there are no specific strategies to improve adherence to intervention protocols. However, as part of the outcome data, we do monitor which treatment, surgical or medical, participants receive.

### Outcome assessment

Assessment of clinical outcomes takes place during outpatient clinic visits at 12, 27, 36, and 60 months after randomisation (PRolaCT-1, PRolaCT-2, and PRolaCT-3), or after study baseline (PRolaCT-O). For PRolaCT-O, study baseline is defined as the moment randomisation would have taken place, thus after all baseline measurements are performed and when treatment is chosen. An extensive overview of enrolment, interventions, and outcome assessment is given in Table 1. During these visits to the outpatient clinic, registration of prolactinoma treatment, physical symptoms, pregnancy, laboratory measurements (including prolactin level), and pituitary MRI (only at 12 and 36 months) takes place. For all surgically treated patients, the final registration of complications takes place during the outpatient clinic visit at 12 months. At every time point, assessment of patient-reported outcome measurements (PROMs) will take place using self-reported questionnaires. These questionnaires assess HRQoL and the presence and burden of physical and psychiatric symptoms or side effects to treatment. In all PRolaCT study arms, PROMs will additionally be collected every 3–6 months after randomisation/study baseline until *T* = 36 for a cost-effectiveness analysis.

**Table 1:**
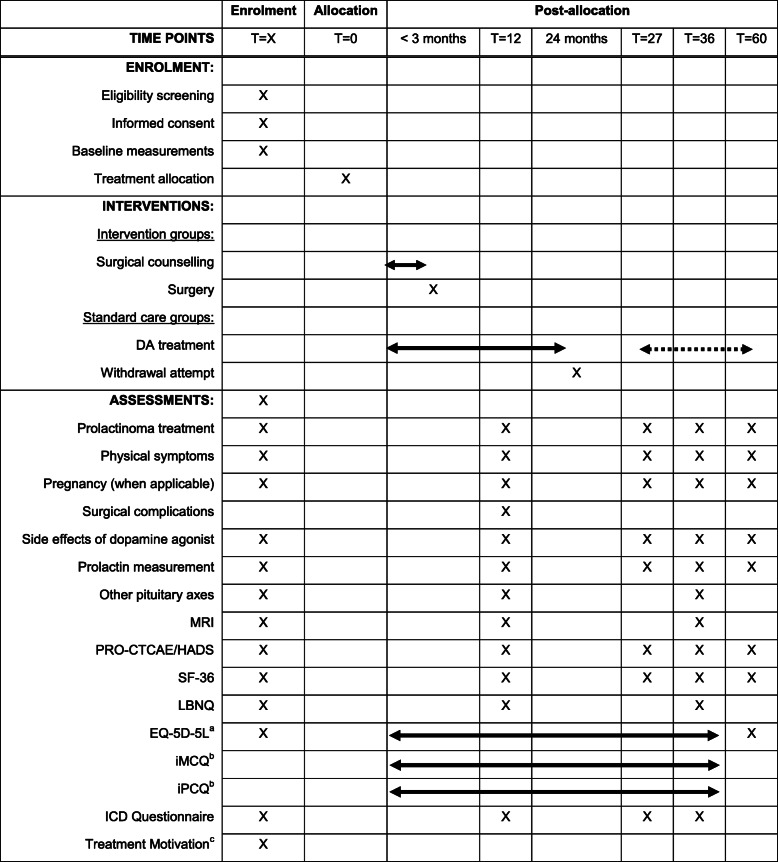
Overview of enrolment, interventions, and outcome assessment

^a^Measured 6, 9, 12, 18, 24, 27, 30, and 36 months after *T* = 0

^b^Measured every 6 months after *T* = 0

^c^Only in PRolaCT-O

### Primary outcomes

The two primary outcomes PRolaCT aims to evaluate are:

1. Health-related quality of life after 12 months of follow-up
2. Disease remission after 36 months of follow-up

#### Health-related quality of life

The primary outcome measure HRQoL is defined as the score on the mental health scale (MHS) of the Medical Outcomes Study (MOS) Short-Form Health Survey (SF-36) after 12 months of follow-up, a time point reflecting a stable situation after surgical treatment or during DA treatment. The SF-36 is the most frequently used questionnaire in prolactinoma patients and has been validated in Dutch and English [22, 31]. The SF-36 is composed of 36 items, organised into eight multi-item scales, including the MHS. The seven other scales assess physical functioning, role limitations due to physical health problems, bodily pain, general health perceptions, vitality, social functioning, and role limitations due to emotional problems [31–33]. In addition to scoring on these scales, the SF-36 also yields two higher order component scores, one for physical health and one for mental health [31–33]. For our primary endpoint, only the score on the MHS will be used, and scores on the other scales and the two component scores will be used for secondary outcome measurements. The MHS score ranges from 0 to 100 and is known to be decreased in patients with active or treated prolactinomas [18–22].

#### Disease remission

Disease remission is defined as the presence of either normoprolactinaemia, which is defined as a prolactin level below the upper limit of normal for the respective laboratory, in the absence of DA treatment for at least 3 months; or pregnancy that was established during at least 3 months of withdrawal of DA treatment. The primary outcome assessment of disease remission is at 36 months of follow-up, reflecting remission after 2 years of DA treatment or long-term remission after surgery.

### Secondary outcomes

The secondary outcomes for this study will be:

- HRQoL measured with the SF-36 (other than the MHS) at baseline and 12, 36, and 60 months after randomisation/baseline
- Disease remission 27 and 60 months after randomisation/baseline
- Biochemical disease control, defined as a normalised prolactin or actual pregnancy 12 months after randomisation/baseline
- Recurrence rate defined as recurrence of hyperprolactinaemia after achieving disease remission, measured 36 and 60 months after randomisation/baseline in all patients who were in remission at the 27-month follow-up time point
- Clinical symptom control, defined as the absence of clinical symptoms, registered in patient’s medical records and measured with the use of the National Cancer Institute Patient-Reported Outcomes version of the Common Terminology Criteria for Adverse Events (PRO-CTCAE) at 12, 27, 36, and 60 months after randomisation/baseline
- Tumour shrinkage on MRI 12 and 36 months after randomisation/baseline
- Pituitary functioning 12, 36, and 60 months after randomisation/baseline
- Complications of surgery registered in the patient’s medical records or side effects to DA treatment, measured with the PRO-CTCAE and a modified Impulse Control Disorder Questionnaire (ICD-Q), 12 and 36 months after randomisation/baseline
- Depression and anxiety scores, measured with the Hospital Anxiety and Depression Scale (HADS), 12 and 36 months after randomisation/baseline
- Disease burden, measured with the Leiden Bother and Needs Questionnaire (LBNQ), 12, 36, and 60 months after randomisation/baseline
- Cost-effectiveness at 12 and 36 months after randomisation/baseline measured with the EQ-5D-5L 6, 9, 12, 18, 24, 27, 30, and 36 months after randomisation/baseline and the iMTA Productivity Cost Questionnaire (iPCQ) and the iMTA Medical Consumption Questionnaire (iMCQ) once every 6 months, starting 6 months after randomisation/baseline, until *T* = 36

### Sample size calculation

To detect a difference in remission rate at 36 months of 40% between surgery (expected rate 90%) and medical treatment (expected rate max. 50%), with a power of 0.8 and an *α* of 0.025, 55 patients per treatment group arm are needed, when considering that max. 30% in the interventional arm will not undergo surgery. However, to detect a difference of 10 points at the SF-36 mental health scale at *T* = 12 with an *α* of 0.025 and a power of 0.8, assuming that the standard deviation is 16, based on the general population [31], and that max. 30% of the interventional arm does not undergo surgery, at least 101 patients per treatment arm are needed. To compensate for the loss to follow-up, it is aimed to include 110 patients per treatment arm and therefore 220 patients in total per RCT/PRolaCT-O (aiming for at least 110 patients per treatment group in PRolaCT-O). If patient recruitment would prove challenging, and the required sample size for separate analyses of the RCTs cannot be reached for two or more of the RCTs (PRolaCT-1, PRolaCT-2, or PRolaCT-3) within the duration of the study, the planned analyses could be performed using the combined sample size for two to three RCTs of 220 patients.

### Planned statistical analyses

We intend to perform all statistical analyses separately for PRolaCT-O and PRolaCT-1, PRolaCT-2, and PRolaCT-3. However, a combined analysis of two to three RCTs may be performed, as stated above, using a regression analysis with adjustment for randomisation group type. A post hoc meta-analysis including all three RCTs and PRolaCT-O will be performed, whatever the course of the study.

To account for positive results due to multiple testing, a lower than usual *p*-value, i.e. < 0.02, will be considered statistically significant. All statistical analyses will be performed according to the intention-to-treat principle. In a sensitivity approach, a per-protocol analysis will also be performed, which may be of added value in case many patients, after consultation, decide not to be operated.

#### Main endpoints

##### Health-related quality of life

In the three RCTs, the difference between the two treatment arms in MHS of the SF-36 will be compared at 12 months using a linear regression analysis, using treatment and the baseline measurement as covariates (analysis of covariance).

##### Disease remission

In the three RCTs, the percentage of patients that achieve disease remission at 36 months will be calculated for the two treatment arms with corresponding 95% confidence interval and compared using a chi-square test.

##### Analyses in PRolaCT-O using propensity score methods

As the treatment groups in PRolaCT-O may differ at baseline, propensity score methods will be used to account for potential confounding. The propensity to receive surgical counselling will be estimated using logistic regression including potential confounders such as duration and effectiveness of prior DA treatment, the amount and severity of side effects to DA treatment measured at study entrance measured with the PRO-CTCAE, age at inclusion, gender (male or female), type of centre treatment takes place (secondary or tertiary), physician enrolling the patient (endocrinologist, gynaecologist, or neurosurgeon), prolactinoma size (micro- or macroadenoma), patient and MDT treatment preference pre- and post-counselling (neutral, medication, or surgery), and the likelihood of positive surgical outcome (unlikely, possibly, or likely) as estimated post hoc by two neurosurgeons blinded to received treatment. Adequacy of the propensity model will be assessed by checking if standardised mean differences after propensity adjustment are below 0.10 and by inspecting balancing plots.

Inverse probability weighting based on the propensity score will be used to balance the treatment groups. After observations are reweighted, treatment groups are compared in the same way as in the RCTs. Propensity matching will be used as a sensitivity analysis.

#### Statistical analyses of secondary outcomes

We will perform several additional analyses of the secondary parameters. When appropriate, we will perform *t*-tests for continuous variables, or regression analysis with adjustment for prognostic baseline variables to increase efficiency and account for suspected confounding. For continuous variables (disease burden, and depression and anxiety scores, for example) measured several times during follow-up and probably missing data of some patients at some time points, longitudinal effects of the treatment strategies will be assessed with linear mixed models. For dichotomous variables, we will calculate differences in risks with a 95% confidence interval and compare the two treatment arms with the use of a chi-square test. Repeated measurements of dichotomous variables will be analysed with generalised estimating equations (if needed with reweighting for missing data) and logistic regression mixed models.

##### Combining the results of the different trials

Since the design of the three RCTs is similar, with the main difference being the duration of DA pre-treatment, the main results will be compared between the three RCTs and the PRolaCT-O study. We first compare the baseline characteristics between the studies. Then, a combined analysis including PRolaCT-O and the RCTs will be performed, using meta-analysis methods. If the results of the different studies differ substantially, we will explore if the differences can be explained by different patient populations in the different studies, using individual patient data (IPD) meta-regression methods, combined with inverse probability weighting.

Moreover, we will compare surgical outcomes between the intervention groups of the three RCTs, using a multivariate analysis, to compare the effects of the duration of DA pre-treatment on surgical outcomes.

In addition to the aforementioned analyses that will be performed according to the intention-to-treat principle, we will perform per-protocol analyses for both our primary and secondary endpoints.

### Economic analysis

#### Cost-effectiveness analysis (CEA)

The economic evaluation will consist of both trial-based cost-effectiveness analyses for all three RCTs and PRolaCT-O (costs per additional patient in remission and cost per quality-of-life adjusted life-year (QALY)) using observed cost and outcome data, and a model-based cost-utility analysis with a lifetime horizon (costs per QALY), in which all different treatment options are compared. In the trial-based economic evaluations, the effects of surgical treatment for patients with prolactinomas will be compared to standard care and related to the difference in costs. Differences in mean costs and effects between strategies will be compared with two-sided bootstrapping. In a net-benefit analysis, costs will be related to the outcomes and presented in a cost-effectiveness acceptability curve. No discounting will be applied due to the short time horizon of the economic evaluation. The evaluation will be performed from a societal perspective. Sensitivity analyses will be carried out for the most important assumptions.

In the model-based cost-utility analysis, a decision tree model will be used to extrapolate the trial results to lifetime costs and QALYs for different surgical treatment options (with and without pre-treatment with cabergoline) in comparison with usual care [29, 30]. In this lifetime cost-utility analysis, costs will be discounted at a percentage of 4% and effects at a percentage of 1.5%, according to the Dutch guidelines for health economic research [34]. Sensitivity analysis will be carried out for the most important input parameters.

Health care use and absence from work will be assessed with the iMCQ and iPCQ. For the valuation of health care, standard prices published in the Dutch costing guidelines will be used [35]. Costs of absenteeism from paid work will be calculated using the friction cost method. QALYs will be estimated with aid of the EQ-5D-5L. Utilities will be calculated from the EQ-5D-5L questionnaire using the Dutch tariff [36]. Using the area-under-the-curve method for the utility scores obtained for each participant, the QALY outcome per participant will be obtained for the trial-based cost-effectiveness analysis. Furthermore, utility values will be used as input in the model for the lifetime cost-effectiveness analysis.

### Data collection

During the study, all study data will be collected and stored in the data management system Castor EDC, with the use of an electronic case record form (eCRF), secured with personal login codes. Participants will be assigned a unique subject number. All data will be collected, stored, and analysed in a coded manner and in accordance with the General Data Protection Regulation (GDPR). The key to subjects’ identification codes will be stored securely at the study site, separate from all other study-related data. After the study ends, all exports of the study data and eCRFs will be stored digitally on the hospital server of the LUMC in a protected folder with access limited to the principal and coordinating investigators of the LUMC. Study data will be stored for 10 years after the study ends. There will be no collection or storing of biological specimens for this study.

### Safety reporting

For the RCTs, any adverse event (AE) that may occur in the standard care groups or the intervention groups after patients are referred back to their own hospital is either an expected result from standard care (and thus measured as part of the study outcomes) or not at all related to the prolactinoma or the received treatment. It is thus highly unlikely that any AE occurring in these patients is related to study participation. AEs will therefore only be recorded for participants in the intervention groups, when they are treated and under medical follow-up in the participating neurosurgical expertise centres. Furthermore, all observed or patient-reported adverse events will be recorded, unless there is a clear relation to pregnancy, e.g. nausea due to morning sickness. All AEs will be followed until they have abated, or until a stable situation has been reached.

All AEs are part of the safety data discussed with the Data Safety Monitoring Board. Moreover, the MREC is notified of all AEs that either result in death, are life-threatening, require non-elective hospitalisation or prolongation of hospitalisation to a maximum of 10 days in case of hospitalisation for prolactinoma surgery, result in persistent or significant disability or incapacity, or are a congenital anomaly or birth defect.

AEs that may occur in participants in PRolaCT-O will not be recorded, because the observational nature of their participation will imply that any AE will not be related to study participation.

### Monitoring

#### Data Safety Monitoring Board (DSMB)

An independent DSMB will perform ongoing safety surveillance for the RCTs. The main responsibility of the DSMB for this study is guarding participant safety. Although complications related to the intervention are uncommon, this is the main interest for the DSMB. No formal interim analyses will be performed.

Monitoring of trial conduct and integrity will be executed every 6 months by internal monitors of the LUMC.

## Ethics and dissemination

The research protocol for the PRolaCT study has been reviewed and approved by the Medical Research Ethics Committee (MREC) of the LUMC and, when appropriate, by the local Institutional Research Boards (IRBs) of all participating centres. There is no formal steering committee or endpoint adjudication committee. However, there is an advisory board consisting of the principal investigators from St. Antonius Ziekenhuis, Medisch Spectrum Twente, and Radboud University Medical Centre, and our patient representative, chair of the Dutch Pituitary Foundation. The advisory board has been consulted during the development phase of the study protocol and is consulted for important protocol modifications. In addition, important protocol modifications are discussed with the DSMB and reviewed by the MREC of the LUMC, as is required by Dutch law on Medical Research involving Human Participants. After MREC approval, all principal investigators, and when deemed appropriate by the MREC, all participants, and the local IRBs will be notified, and the trial registry will be updated. Important protocol modifications will be declared transparently in the publication of the primary results.

Standard of care, which is primarily assessed in this study, is being covered by regular patient insurance. Damages to research participants through injury or death caused by the study will be reimbursed through a standard insurance for research participants, which covers the damage that becomes apparent during the study, or within 4 years after the end of the study. The risk of injury or death due to study interventions, which is neurosurgical counselling, is virtually non-existent.

After completion of the trials, the primary results of the RCTs will be unreservedly published in an open access and peer-reviewed journal.

## Discussion

### Rationale for the study design

Prolonged DA treatment for prolactinoma, which is current standard practice [1, 4, 5], is very effective in controlling hyperprolactinaemia and restoring gonadal function. However, patients may suffer from substantial side effects compromising HRQoL to a greater extent than previously described, while remission rates following DA treatment are lower than previously thought [5–13, 18–22, 37]. Although there is a low risk of complications, surgical resection induces swift disease remission in most patients, which may allow a drug-free future [5, 17, 23–26, 28, 38–46] with improved or even normalised HRQoL, and may thus deserve a more prominent place in the treatment of prolactinoma patients. However, loss of pituitary function and life-threatening complications must be considered.

The PRolaCT studies combine three randomised clinical trials and an observational cohort and are designed to compare both treatment strategies for patient-relevant outcomes, such as disease remission and HRQoL. For the RCT parts of PRolaCT, patients consent to randomisation between surgical counselling or standard care. If we would randomise between surgery versus DA treatment, all patients would have had to be referred to a neurosurgical expertise centre for surgical counselling prior to entering the study. With our pragmatic RCT design, only patients who randomise for surgical counselling are referred to the regional MDT to receive detailed information about surgical treatment for prolactinoma and standard care is delivered in the regional hospital. Patients who randomise for standard care are thus not burdened with referral to a neurosurgical expertise centre and neurosurgical counselling. Although a greater appeal on regional networks is made, the pragmatic design of PRolaCT intends to lower the threshold for randomisation.

The study intervention in PRolaCT is thus the neurosurgical counselling rather than surgical intervention, which allows patients in the intervention groups to opt for surgery. If there is a substantial proportion of patients in the intervention group who do not undergo surgery, it would lead to an attenuation of found effects. Furthermore, as a part of standard care, participants in the control groups may still undergo surgery in the follow-up of the study (which is sporadically needed in case of severe DA intolerance or resistance). The expected cross-over between treatments was taken into account for the sample size calculation, resulting in a larger sample size needed. However, we anticipate that neurosurgical counselling greatly contributes to patients’ decision for a specific treatment, and therefore, our approach may decrease the risk of study drop-out due to disappointment over the outcome of randomisation.

### Strong patient preference

In our experience, many prolactinoma patients are willing to undergo surgery, which was confirmed by a recent survey conducted by the Dutch Pituitary Foundation. Nonetheless, patients’ willingness to be randomised appears diminished due to a strong preference for a specific treatment, which was generally, but not exclusively, for surgical resection, and patient recruitment has thus far been limited. To overcome this impaired patient recruitment, PRolaCT-O primarily aims to compare HRQoL, remission rates, and cost-effectiveness of neurosurgical and medical prolactinoma treatment in patients who are eligible to participate in one of the RCTs, but refuse randomisation.

The non-interventional nature of PRolaCT-O has some limitations, mainly based on the assumption that all confounders are known and adequately measured. For PRolaCT-O, confounding by indication can be expected, but comprehensive baseline measurements, including exploration of patients’ and physicians’ motivation for a specific treatment choice, may allow for correction for expected confounders, such as the expected likelihood of complete resection, the effectiveness of previous DA treatment, or the amount and severity of side effects to DA treatment at study baseline. Nonetheless, reviews comparing observational studies and RCTs have found effect estimate differences, although not all statistically significant, even when correction for confounding by propensity scoring was used [47, 48]. However, these differences may as well be explained by varying outcome definitions, differentially distributed effect modifiers in the underlying population, when estimates of population-averaged effects are used, and bias in both RCTs and observational studies. Therefore, found differences between effect estimates of observational and randomised studies do not necessarily mean observational studies are less reliable [49]. Clearly, PRolaCT-O cannot replace the randomised studies that are and will remain the mainstay of PRolaCT. However, PRolaCT-O, which aims to have a lower patient threshold for participation, might be more easily implemented in clinical practice, and we believe will provide study results earlier than the RCTs.

Collection of “real-world” data with PRolaCT-O might provide interesting insights in patient characteristics (e.g. presence and severity of symptoms or side effects, prolactinoma characteristics), which may be used for clinical decision-making, facilitating more individually tailored practice of evidence-based medicine.

## Trial status

Current protocol version 5, issue date 7 Jan 2021. Recruitment started June 21, 2019, and is planned to be completed in April 2022.

## Supplementary Information

**Additional file 1.** SPIRIT checklist

**Additional file 2.** First MERC approval protocol dated 12 March 2019 (original Dutch)

**Additional file 3.** First MERC approval protocol dated 12 March 2019 (English translation)

**Additional file 4.** MERC approval amendment PRolaCT-O dated 21 January 2020 (original Dutch)

**Additional file 5.** MERC approval amendment PRolaCT-O dated 21 January 2020 (English translation)

**Additional file 6.** Grant ZonMw dated 29 June 2017 (original Dutch)

**Additional file 7.** Grant ZonMw dated 29 June 2017 (English translation)

## Data Availability

The complete trial dataset will be stored by and accessible to the coordinating centre (LUMC). All principal investigators from the participating centres will have access to datasets containing only participants from their own centres. Analysed datasets will be available from the corresponding author upon reasonable request.

## Abbreviations

CEA: Cost-effectiveness analysis
DA: Dopamine agonist
DSMB: Data safety monitoring board
eCRF: Electronic case record form
eGFR: Glomerular filtration rate
EDC: Electronic data capture
GDPR: General data protection regulation
HADS: Hospital anxiety and depression scale
HRQoL: Health-related quality of life
ICD: Impulse control disorder
ICD-Q: Impulse control disorder questionnaire
IPD: Individual patient data
IRB: Institutional research board
LBNQ: Leiden bother and needs questionnaire
MCQ: Medical consumption questionnaire
MHS: Mental health scale
MREC: Medical research ethics committee
MRI: Magnetic resonance imaging
PCQ: Productivity cost questionnaire
PRO-CTCAE: Patient-reported outcomes version of the common terminology criteria for adverse events
PROM: Patient-reported outcome measurement
QALY: Quality-adjusted life years
RCT: Randomised clinical trial
SF-36: Short-Form Health Survey
SPIRIT: Standard protocol items: recommendations for interventional trials
