# Supplementary material for: The PRolaCT studies — a study protocol for a combined randomised clinical trial and observational cohort study design in prolactinoma
Source: Trials. 2021 Sep 25;22:653. doi: 10.1186/s13063-021-05604-y (PMC8465768; doi:10.1186/s13063-021-05604-y)
Supplement: Supplementary file 2 — Additional file 2. First MERC approval protocol dated 12 March 2019 (original Dutch) [file 13063_2021_5604_MOESM2_ESM.pdf]

Leids Universitair
Medisch Centrum

afdeling Commissie Medische Ethiek
postzone P5-P
Mw. mr. S.W.C. Bonnet

telefoon (071) 526 3241 of (071) 5266963
e-mail cme@lumc.nl
onze referentie **P18.219**/SB/sb
uw referentie
Ccmo ref NL63919.058.18
datum 12 maart 2019
onderwerp **Besluit tot positief oordeel CME**

aan De hooggeleerde vrouwe
prof. dr. N.R. Biermasz

afdeling Endocrinologie / AIG

postzone **C7-Q, alhier**

Geachte mevrouw Biermasz,

Hierbij zend ik u het besluit van de Commissie Medische Ethiek (CME) van het LUMC inzake het onderzoeksprotocol getiteld: **"Prolact - three multicenter prolactinoma randomized clinical trials" (NL63919.058.18)**.

De CME verleent goedkeuring aan genoemd onderzoek. Voor de overwegingen verwijs ik u naar het bijgevoegde besluit.

De CME wijst u op de verplichtingen die voortvloeien uit de WMO en de aanverwante regelgeving, waarvan een overzicht is opgenomen in de bijlage behorend bij dit besluit.

Verder wijst zij u erop dat definitieve toestemming van de Raad van Bestuur nodig is voordat tot uitvoering van het onderzoek kan worden overgegaan. De Commissie zal de Raad van Bestuur van het LUMC in kennis stellen van haar oordeel.

Tenslotte verzoeken wij u alle bij de uitvoering van het onderzoek betrokken partijen van het besluit op de hoogte te brengen

Vertrouwend u hiermee voldoende te hebben geïnformeerd.

Met vriendelijke groet,
namens de Commissie Medische Ethiek,

Mw. mr. S.W.C. Bonnet
secretaris

cc: Raad van Bestuur van het LUMC
WeCieHAIG@lumc.nl, Endocrinologie / AIG, LUMC, Leiden
dr. W.R. van Furth, Neurochirurgie, LUMC, Leiden
drs. I.M. Zandbergen, Neurochirurgie, LUMC, Leiden
drs. M.A. Schroijen, Endrocrinologie, LUMC, Leiden
CCMO d.m.v. upload in Toetsing OnLine (NL63919.058.18)

L U
M C Leids Universitair Medisch Centrum

BESLUIT
Primaire beoordeling

NL nummer	NL63919.058.18	CME nummer	P18.219
Titel onderzoek	"Prolact - three multicenter prolactinoma randomized clinical trials"		

Contactgegevens: prof. dr. N.R. Biermasz, Endocrinologie / AIG, LUMC, Leiden
Verrichter: LUMC, Leiden

Besluit
De Commissie Medische Ethiek (CME) van het LUMC heeft zich, op grond van artikel 2, tweede lid, sub a van de Wet Medisch-wetenschappelijk Onderzoek met mensen (WMO), beraden over bovenstaand onderzoeksdossier.

De CME oordeelt positief over het onderzoeksdossier uit te voeren in de volgende centra:

- het LUMC te Leiden (hoofdonderzoeker: Prof. dr. N.R. Biermasz en dr. W.R. van Furth)
- Reinier de Graaf Groep (hoofdonderzoeker: dr. C.J. Kapiteijn)

Documenten
Het oordeel is gebaseerd op de documenten die in bijlage 1 zijn vermeld.

Achtergrond
Op 18-10-2018 is het onderzoeksdossier ter beoordeling bij de CME ingediend en in behandeling genomen. Het onderzoeksdossier is besproken in de vergadering van 27 november 2018; zie bijlage 2 voor de samenstelling van de Commissie ten tijde van deze vergadering. Voor de beoordeling van enkele kleine wijzigingen, is het dossier vervolgens nog besproken in de vergadering van het Dagelijks Bestuur van de CME van 19 februari 2019.

Overwegingen
De CME is van oordeel dat aan de voorwaarden in artikel 3 a t/m m van de WMO is voldaan. De belangrijkste vragen hadden betrekking op het toevoegen van de complicatie "verlies van reuk en dus van smaak", gegevensbescherming, het verduidelijken van de procedure gerelateerde complicaties in het protocol (DSMB), de tekst van de informatiefolder, het ontbreken van een getekend onderzoekscontract alsmede onvolkomenheden in de aan de Commissie ter beoordeling voorgelegde documenten. Nadat de documenten op een juiste wijze zijn aangepast, de vragen naar tevredenheid zijn beantwoord en het getekende onderzoekscontract is overgelegd, is besloten de beoordeling af te ronden met een positief besluit.

De CME heeft de in bijlage 1 vermelde onderzoeksverklaring bekeken. Zij heeft geconstateerd dat is voldaan aan de voorwaarden in artikel 3, onderdeel f, van de WMO.

De CME is van oordeel dat het onderzoeksprotocol in een toestemmingsprocedure voorziet die overeenstemt met artikel 6, eerste en derde lid, van de WMO.

Albinusdreef 2, Postbus 9600, 2300 RC Leiden

De CME is van mening dat is voldaan aan de voorwaarden in artikel 6, vijfde t/m negende lid, van de WMO. De proefpersonen worden op gepaste, volledige en begrijpelijke wijze schriftelijk over het onderzoek geïnformeerd en over de mogelijkheid om de toestemming te allen tijde in te trekken.

Verzekeringen
De CME heeft geconstateerd dat is voldaan aan de verzekeringsplicht. Er is een proefpersonenverzekering afgesloten zoals bepaald in artikel 7, eerste lid, van de WMO en zoals nader uitgewerkt in het Besluit verplichte verzekering bij medisch-wetenschappelijk onderzoek met mensen (Besluit van 24 november 2014).

Het onderzoek valt onder de proefpersonenverzekering van het LUMC afgesloten bij Centramed.

De CME heeft geconstateerd dat een aansprakelijkheidsverzekering is afgesloten zoals bepaald in artikel 7, negende lid, van de WMO.

Ten slotte wijst de CME u op de voorwaarden en verplichtingen die in bijlage 3 zijn vermeld.

Hoogachtend,
Namens de Commissie Medische Ethiek van het LUMC,

Mw. mr. S.W.C. Bonnet,
secretaris

Leiden, 12 maart 2019

Beroepsprocedure
Tegen dit besluit kan een belanghebbende op grond van artikel 23 van de WMO binnen zes weken na de dag waarop het besluit is bekend gemaakt, administratief beroep instellen bij de Centrale Commissie Mensgebonden Onderzoek (CCMO). Het beroepschrift dient u te adresseren aan CCMO, Postbus 16302, 2500 BH Den Haag.

Albinusdreef 2, Postbus 9600, 2300 RC Leiden

Leids Universitair Medisch Centrum

Bijlage 1

Documenten

- A1 Aanbiedingsbrief d.d. 16-10-2018; aanbiedingsmail d.d. 18-10-2018
- A1 Ontvangstbevestiging dossier niet volledig d.d. 24-10-2018
- A1 Antwoordmail indiener ontbrekende documenten d.d. 31-10-2018
- A1 Vraagbrief METC d.d. 06-12-2018; nadere vraagmail METC d.d. 06-12-2018
- A1 Antwoordbrief indiener d.d. 01-02-2019; antwoordmail indiener d.d. 08-02-2019
- A1 Nadere vraagbrief METC per mail d.d. 04-03-2019
- A1 Antwoordmail indiener d.d. 06-03-2019
- B1 ABR-formulier versie 03 d.d. 06-03-2019
- C1 Protocol versie 1.2 d.d. 04-03-2019
- D2 SPC Cabergoline Sandoz (RVG 105105) per 18-11-2016
- D2 SPC Bromocriptine Parlodel (RVG 08202 en RVG 09355) per 02-03-2018
- D2 SPC Cabergoline Dostinex (RVG 15375) per 25-10-2015
- D2 SPC Quinagolide Norprolac (RVG 16289 en RVG 16290) per 21-03-2016
- D2 SPC Cabergoline PCH (RVG 34176) per 02-04-2015
- D2 SPC Cabergoline Aurobindo (RVG 34200) per 04-10-2017
- E1 Informatiebrief proefpersonen PRolaCT-1 versie 1.1 d.d. 03-01-2019
- E1 Informatiebrief proefpersonen PRolaCT-2 versie 1.1 d.d. 03-01-2019
- E1 Informatiebrief proefpersonen PRolaCT-3 versie 1.1 d.d. 03-01-2019
- E2 Toestemmingsformulier proefpersonen versie 1.1 d.d. 07-12-2018
- E4 Informatiefolder - de Behandeling van een Prolactinoom versie 1.0 d.d. 04-10-2018
- E4 Fact sheet patiëntvoorlichting versie 1.0 d.d. 21-09-2018
- E4 Informatiefolder proefpersonen versie 1.1 d.d. 03-01-2019
- F1 Hospital Anxiety and Depression Scale (HADS)
- F1 iMTA Medical Consumption Questionnaire (iMCQ)
- F1 Leiden Bother & Needs Questionnaire (LBNQ)
- F1 SF-36
- F1 EQ-5D-5L
- F1 PRO-CTCAE versie 1.0 d.d. 10-10-2018
- F1 Impulse Control Disorder Questionnaire (ICD-Q) versie 1.0 d.d. 10-10-2018
- F1 Work Role Functioning Questionnaire (WRFQ)
- G1 Certificaat proefpersonenverzekering LUMC, polisnummer 624.530.305, CentraMed d.d. 01-2019
- G2 Bewijs dekking Aansprakelijkheidsverzekering LUMC, polisnummer 620.872.908, CentraMed d.d. 01-2019
- H1 CV onafhankelijk deskundige mw. drs. M.A. Schroijen
- H2 CV coördinerend onderzoeker mw. drs. I.M. Zandbergen
- I1 Lijst van deelnemende centra versie 1.0 d.d. 16-10-2018
- I2 Onderzoeksverklaring RdGG d.d. 03-09-2018
- I3 CV hoofdonderzoeker RdGG mw. dr. K. Kapiteijn
- I3 CV hoofdonderzoeker LUMC dr. W.R. van Furth d.d. 24-10-2018

Albinusdreef 2, Postbus 9600, 2300 RC Leiden

- I3 CV hoofdonderzoeker LUMC dr. N.R. Biermasz
- K1 Goedkeuring wetenschapscommissie Interne Geneeskunde d.d. 30-08-2018
- K1 Goedkeuring wetenschapscommissie Neurochirurgie d.d. 31-10-2018
- K3 Onderzoekscontract PRolaCT d.d. 25-02-2019
- K3 Verklaring referentiecontract d.d. 06-03-2019
- K5 DSMB Charter versie 1.1 d.d. 08-02-2019
- K6 Risicoclassificatie matig versie 1.0 d.d. 03-09-2018
- K6 Model Brief aan huisarts versie 1.0 d.d. 08-02-2019

Albinusdreef 2, Postbus 9600, 2300 RC Leiden

Leids Universitair
Medisch Centrum

Bijlage 2

Samenstelling Commissie Medische Ethiek van het LUMC

Prof. dr. A. Dahan,	Voorzitter, Arts
Dr. U.A. Badrising	Arts
Mw. K. Bus	Proefpersonenlid
Dr. mr. M.M. Eijkholt	Jurist
Dr. ir. S.J.P.M. van Engelen	Deskundige op het gebied van Medical Devices
Dr. R.H.H. Groenwold	Methodoloog
Prof. dr. H.J. Guchelaar	Ziekenhuisapotheker en Klinisch farmacoloog
Dr. M.C. Haak	Arts
Dr. M. Houtlosser	Ethicus
Drs. P. van Houwelingen	Proefpersonenlid
Dr. H.W. Kapiteijn	Arts
Mw. C.C. Kliphuis	Proefpersonenlid
Dr. G.J. Liefers	Arts
Prof. dr. E. Lopriore	Kinderarts
Mr. M.F. van der Mersch	Jurist
Dr. A.B. te Pas	Kinderarts
Mr. C.E. Philips-Santman	Jurist
Prof. dr. H. Putter	Methodoloog
Dr. C.G. Reichart	Arts
Dr. A.J.H.A. Scholte	Arts
Dr. J.J. Swen	Ziekenhuisapotheker en Klinisch Farmacoloog
Dr. D.P. Touwen	Medisch Ethicus
Dr. M.E. Tushuizen	Arts
Drs. Y. In 't Veld	Proefpersonenlid
Dr. M. van Velzen	Deskundige op het gebied van onderzoek
Prof. dr. M.C. de Vries	Arts en Medisch Ethicus (vice-voorzitter)
Prof. dr. M.J.H. Wermer	Arts
Dr. J. Zwaveling	Ziekenhuisapotheker en Klinisch farmacoloog
Dr. E.W. van Zwet	Methodoloog

Albinusdreef 2, Postbus 9600, 2300 RC Leiden

Bijlage 3

Voorwaarden en verplichtingen

Geen bezwaar bevoegde instantie
Er kan pas met het onderzoek worden gestart, wanneer eveneens geen bezwaar wordt gemaakt binnen de voorgeschreven termijn door de bevoegde instantie. Deze verklaring is door de CME ontvangen op d.d.

Geldigheid oordeel
Het positieve oordeel verliest zijn geldigheid als de inclusie van de eerste proefpersoon niet heeft plaatsgevonden binnen een jaar nadat dit besluit is genomen.

Amendementen
Amendementen dienen ter beoordeling aan de CME te worden voorgelegd.

Startdatum onderzoek
De CME dient op de hoogte te worden gesteld van de definitieve startdatum van het onderzoek. Dat is de datum waarop de inclusie van de eerste proefpersoon plaatsvindt.

Voortgangsrapportage
Eén jaar na datum van het oordeel, en ieder jaar daaropvolgend, dient de METC op de hoogte te worden gebracht van de voortgang van de studie middels het formulier Voortgangsrapportage.

Melding SAE's
SAE's dienen aan de CME te worden gemeld.

Advies DSMB
Indien een advies van de DSMB niet volledig wordt opgevolgd, dient de CME het advies met toelichting over het niet (volledig) opvolgen van het advies te ontvangen en toestemming te geven voor voortzetting van het onderzoek.

Melding (voortijdige) beëindiging
(Voortijdige) beëindiging van het onderzoek dient, met redenen omkleed, te worden gemeld aan de CME.

Eindrapportage
De CME dient op de hoogte te worden gebracht van de resultaten van het onderzoek middels een eindrapport.

Termijnen en overige uitleg ten aanzien van de indiening van de verschillende documenten aan de CME *vindt u op de website van de* CCMO *bij het standaard onderzoeksdossier en de toelichting daarop.*

Albinusdreef 2, Postbus 9600, 2300 RC Leiden
