# Supplementary material for: The PRolaCT studies — a study protocol for a combined randomised clinical trial and observational cohort study design in prolactinoma
Source: Trials. 2021 Sep 25;22:653. doi: 10.1186/s13063-021-05604-y (PMC8465768; doi:10.1186/s13063-021-05604-y)
Supplement: Supplementary file 3 — Additional file 3. First MERC approval protocol dated 12 March 2019 (English translation) [file 13063_2021_5604_MOESM3_ESM.pdf]

Our reference: P18.219
CCMO reference: NL63919.058.12
Date: 12th march 2019
Subject: approval by CME

Dear Mrs. Biermasz,

Herewith I send you the decree from the Committee Medical Ethics (CME) of the LUMC regarding the study protocol entitled: **"Prolact – three multicenter prolactinoma randomized clinical trials" (NL639191.058.18)**.

The CME grants her permission for execution of named study. For our considerations I refer you to the enclosed decision.

The CME points out to you, the obligations arising from the WMO *(Law on Medical scientific research involving humans; in Dutch: Wet Medisch-Wetenschappelijk Onderzoek met mensen)* and related regulations, of which an overview has been enclosed in the appendix accompanying this decree.

Furthermore, the committee points out that definitive permission by the Executive Board is needed before one can proceed with execution of the study. The committee shall notify the Executive Board of her judgement.

Lastly, we request you notify all parties involved with the study of the decision.

We trust that we have sufficiently informed you.

Yours sincerely,
on behalf of the Committee Medical Ethics,

Mrs. mr. S.W.C. Bonnet
Secretary

DECREE
Primary judgement

NL number	NL63919.058.18	CME number	P18.219
Title study	"Prolact – three multicenter prolactinoma randomized clinical trials"		

Contact information: prof. dr. N.R. Biermasz, Endocrinology / AIG, LUMC, Leiden
Sponsor: LUMC, Leiden

Decree
The Committee Medical Ethics (CME) of the LUMC has considered abovementioned research file based on article 2, section two, sub a of the Law on Medical scientific research involving humans (in Dutch: Wet Medisch-Wetenschappelijk Onderzoek met mensen; WMO).

The CME approves execution of the study file in the following centers:

- The LUMC in Leiden (Principal Investigator: prof. dr. N.R. Biermasz and dr. W.R. van Furth)
- Renier de Graaf Groep (Principal Investigator: dr. C.J. Kapiteijn)

Documents
The judgement was based on the documents mentioned in appendix 1.

Background
18 October 2018, the research proposal has been submitted for approval, and has been taken into consideration by the CME. The research file has been discussed in the meeting of 27 November 2018; see appendix 2 for composition of the Committee at the time of this meeting. For consideration of some minor revisions, the dossier has subsequently been discussed in the meeting of the Daily Board of the CME on 19 February 2019.

Considerations
The CME judges that this study adheres to article 3 a to m of the WMO. The most important questions regarded addition of complication "loss of smell and thereby taste", data protection, clarification of the procedure relating complication in the protocol (DSMB), the text of the information leaflet, lack of a signed clinical trial agreement, and imperfection of the documents submitted to the Committee. After rightful revision of the documents, the questions were answered sufficiently, and a signed clinical trial agreement has been submitted, a positive decision was reached.

The CME has reviewed the in appendix 1 named research statement. She has observed that this adheres to the conditions in article 3, section f, of the WMO.

The CME judges that the research protocol provides a consent procedure that agrees with article 6, sections one and three, of the WMO.

The CME judges that the conditions in article 6, section five to nine, of the WMO, are met. Research subjects are appropriately, completely, and understandably informed in writing about the study, and the possibility to withdraw consent.

Insurance

The CME has observed that the insurance obligation is met. A subject insurance has been taken out as defined in article 7, section one, of the WMO and as further specified in the Decree mandatory insurance in medical-scientific research involving humans (Decree of 24 November 2014).

The study falls under the scope of the research subject insurance of the LUMC taken out with Centramed.

The CME has observed that a liability insurance has been taken out as determined in article 7, section nine, of the WMO.

Lastly, the CMO points out to you the conditions and obligations named in appendix 3.

Yours sincerely,
on behalf of the Committee Medical Ethics,

Mrs. mr. S.W.C. Bonnet
Secretary

APPENDIX 1
Documents

- A1. Cover letter dated 16-10-2018; accompanying email dated 18 Oct 2018
- A1. Confirmation of receipt incomplete file dateddated 24 Oct 2018
- A1. Answering email submitter missing documents dateddated 31 Oct 2018
- A1. Question letter METC dated 06-12-2018; follow-up question email METC dated 6 Dec 2018
- A1. Answering letter submitter dated 01-02-2019; answering email submitter dated 8 Feb 2019
- A1. Follow-up question letter METC per email dated 4 Mar 2019
- A1. Answering email submitter dated 6 Mar 2019
- B1. ABR-form version 03 dated 6 Mar 2019
- C1. Protocol version 1.2 dated 4 Mar 2019
- D2. SPC Cabergoline Sandoz (RVG 105105) per 18 Nov 2016
- D2. SPC Bromocriptine Parlodel (RVG 08202 en RVG 09355) per 2 Mar 2018
- D2. SPC Cabergoline Dostinex (RVG 15375) per 25 Oct 2015
- D2. SPC Quinagolide Norprolac (RVG 16289 en RVG 16290) per 21 Mar 2016
- D2. SPC Cabergoline PCH (RVG 34176) per 2 Apr 2015
- D2. SPC Cabergoline Aurobindo (RVG 34200) per 4 Oct 2017
- E1. Subject information letter PRolaCT-1 version 1.1 dated 3 Jan 2019
- E1. Subject information letter PRolaCT-2 version 1.1 dated 3 Jan 2019
- E1. Subject information letter PRolaCT-3 version 1.1 dated 3 Jan 2019
- E2. Subject informed consent form version 1.1 dated 7 Dec 2018
- E4. Information leaflet – Treatment of a prolactinoma version 1.0 dated 4 Oct 2018
- E4. Fact sheet patient information version 1.0 dated 21 Sep 2018
- E4. Information leaflet version 1.1 dated 3 Jan 2019
- F1. Hospital Anxiety and Depression Scale (HADS)
- F1. iMTA Medical Consumption Questionnaire (iMCQ)
- F1. Leiden Bother &Needs Questionnaire (LBNQ)
- F1. SF-36
- F1. EQ-SD-SL
- F1. PRO-CTCAE version 1.0 dated 10 Oct 2018
- F1. Impulse Control Disorder Questionnaire (ICD-Q) version 1.0 dated 10 Oct 2018
- F1. Work Role Functioning Questionnaire (WRFQ)
- G1. Certificate study subject insurance LUMC, policy number 624.530.305, CentraMed dated Jan 2019
- G2. Evidence of coverage liability insurance LUMC, policy number 620.872.908, CentraMed dated Jan 2019
- H1. CV independent expert mrs. M.A. Schroijen M.D.

- H2. CV coordinating investigator mrs. I.M. Zandbergen M.D.
- I1. List of participating centers 1.0 dated 16 Oct 2018
- I2. Research decralation RdGG dated 03 Sep2018
- I3. CV principal investigator RdGG mrs. K. Kapiteijn M.D.
- I3. CV principal investigator LUMC mr. W.R. van Furth M.D., PhD dated 24 Oct 2018
- I3. CV principal investigator LUMC mrs. N.R. Biermasz M.D., PhD
- K1. Approval science committee internal medicine dated 30 Aug 2018
- K1. Approval science committee neurosurgery dated 31 Oct 2018
- K3. Clinical trial agreement PRoIaCT dated 25 Feb 2019
- K3. Declaration reference agreement dated 6 Mar 2019
- K5. DSMB Charter version 1.1 dated 8 Feb 2019
- K6. Risk classification version 1.0 dated 3 Sep 2018
- K6. Model letter to family doctor version 1.0 dated 8 Feb 2019

APPENDIX 2
Composition Committee Medical Ethics of the LUMC

Prof. dr. A. Dahan,	Chairman, doctor
Dr. U.A. Badrising	Doctor
Mrs. K. Bus	Patient representative
Dr. mr•. M.M. Eijkholt	Lawyer
Dr. ir. S.J.P.M. van Engelen	Expert in the field of Medical Devices
Dr. R.H.H. Groenwold	Methodologist
Prof. dr. H.J. Guchelaar	Hospital pharmacist and clinical farmacologist
Dr. M.C. Haak	Doctor
Dr. M. Houtlosser	Ethicist
Drs. P. van Houwelingen	Patient representative
Dr. H.W. Kapiteijn	Doctor
Mrs. C.C. Kliphuis	Patient representative
Dr. G.J. Liefers	Doctor
Prof. dr. E. Lopriore	Pediatric doctor
Mr. M.F. van der Mersch	Lawyer
Dr. A.B. te Pas	Pediatric doctor
Mr. C.E. Philips-Santman	Lawyer
Prof. dr. H. Putter	Methodologist
Dr. C.G. Reishart	Doctor
Dr. A.J.H.A. Scholte	Doctor
Dr. J.J. Swen	Hospital pharmacist and clinical farmacologist
Dr. D.P. Touwen	Medical ethicist
Dr. M.E. Tushuizen	Doctor
Drs. Y. In 't Veld	Patient representative
Dr. M. van Velzen	Expert in the field of research
Prof. dr. M.C. de Vries	Doctor and medical ethicist (vice-chairman)
Prof. dr. M.J.H. Wermer	Doctor
Dr. J. Zwaveling	Hospital pharmacist and clinical farmacologist
Dr. E.W. van Zwet	Methodologist

APPENDIX 3
Conditions and obligations

No objection from competent authority
The study cannot start until there is no objection from the competent authority within the regulatory period. This declaration has been received by the CME on …

Validity of the judgement
The positive judgement loses its validity if the first patient has not been included in the study within one year after the judgement was made.

Amendments
Amendments should be submitted to the CME for approval.

Date of study start
The CME should be informed about the definitive start date of the study. This is the date that the first patient is included.

Progress report
One year after approval, and once yearly thereafter, the METC should be informed about the study progress, by means of the progress report form.

Report of SAEs
SAEs should be reported to the CME.

DSMB advice
In case a DSMB advice is not completely followed, the CME should receive the advice and an explanation of not (completely) following the advice, and approve the continuation of the study.

Reporting of (preliminary) termination
(Preliminary) termination of the study should be reported to the CME, including rationale.

Final report
The CME should be informed about the study results in a final report.

Terms and other explanations regarding submission of various document to the CME can be found on the website of the CCMO, section on the standard research file and the corresponding elucidation.
