# Supplementary material for: The PRolaCT studies — a study protocol for a combined randomised clinical trial and observational cohort study design in prolactinoma
Source: Trials. 2021 Sep 25;22:653. doi: 10.1186/s13063-021-05604-y (PMC8465768; doi:10.1186/s13063-021-05604-y)
Supplement: Supplementary file 4 — Additional file 4. MERC approval amendment PRolaCT-O dated 21 January 2020 (original Dutch) [file 13063_2021_5604_MOESM4_ESM.pdf]

Medisch-Ethische Toetsingscommissie
Leiden | Den Haag | Delft

commissie METC LDD
postzone P5-P
Mw. P.A. Visser

telefoon (071) 526 3241 Of (071)5266963
e-mail metc-ldd@lumc.nl

onze referentie **P18.219**/PV/pv
uw referentie
Ccmo ref NL63919.058.18
datum 21 januari 2020
onderwerp **Besluit beoordeling amendement NL63919.058.18**

aan De hooggeleerde vrouwe
prof. dr. N.R. Biermasz

afdeling Endocrinologie / AIG

postzone **C7-Q, alhier**

Geachte mevrouw Biermasz,

Hierbij zend ik u het besluit van de medisch-ethische toetsingscommissie Leiden Den Haag Delft (METC LDD) inzake het onderzoeksprotocol getiteld: **"Prolact - three multicenter prolactinoma randomized clinical trials" (NL63919.058.18)**.

De METC LDD verleent goedkeuring aan het amendement in genoemd onderzoek. Voor de overwegingen verwijs ik u naar het bijgevoegde besluit.

Wij verzoeken u alle bij de uitvoering van het onderzoek betrokken partijen van het besluit op de hoogte te brengen

Vertrouwend u hiermee voldoende te hebben geïnformeerd.

Met vriendelijke groet,
namens de METC Leiden Den Haag Delft,

Mw. P.A. Visser
Secretaris

cc: WeCieHAIG@lumc.nl, Endocrinologie / AIG, LUMC, Leiden
drs. I.M. Zandbergen, Neurochirurgie, LUMC, Leiden
CCMO d.m.v. upload in ToetsingOnline (NL63919.058.18)

BESLUIT
Beoordeling amendement

NL nummer	NL63919.058.18	METC-nummer	P18.219
Titel onderzoek	Prolact - three multicenter prolactinoma randomized clinical trials		

Contactgegevens: prof. dr. N.R. Biermasz, Endocrinologie / AIG, LUMC, Leiden
Verrichter: LUMC, Leiden

Besluit
De medisch-ethische toetsingscommissie Leiden Den Haag Delft (METC LDD) heeft zich, op grond van artikel 2, tweede lid, sub a van de Wet medisch-wetenschappelijk onderzoek met mensen (WMO), beraden over het amendement behorend bij bovengenoemd onderzoeksdossier.

De METC LDD heeft eerder een positief oordeel gegeven over het onderzoeksdossier in de volgende centra:
- LUMC te Leiden (hoofdonderzoeker: prof. dr. N.R. Biermasz)
- Amsterdam UMC, locatie AMC, te Amsterdam (hoofdonderzoeker: dr. J. Hoogmoed)
- Amsterdam UMC, locatie VUmc, te Amsterdam (hoofdonderzoeker: prof. dr. M.L. Drent)
- Elisabeth-Tweesteden Ziekenhuis te Tilburg (hoofdonderzoeker: dr. B. Burhani)
- Radboudumc te Nijmegen (hoofdonderzoeker: dr. H.D. Boogaarts)
- Reinier de Graaf Groep (hoofdonderzoeker: dr. C.J. Kapiteijn)

De commissie oordeelt positief over het amendement.

Documenten
Het besluit is gebaseerd op de documenten die in bijlage 1 zijn vermeld.

Achtergrond
Op 17-10-2019 is het amendement ter beoordeling bij de METC LDD ingediend. Het amendement heeft betrekking op de toevoeging van een observationele onderzoeksarm de PRolaCT-O, wijziging van de exclusiecriteria, wijziging in de inclusieprocedure en wijziging in de registratie van *adverse events*. Genoemde zaken zijn bij besluit van 15-11-2019 goedgekeurd. Aansluitend hierop zijn de proefpersoneninformatiebrieven aangepast en is een informatiebrief toegevoegd voor de PRolaCT-0 onderzoeksarm. Deze informatiebrieven heeft de commissie nu beoordeeld.

Het amendement is beoordeeld door het proefpersonenlid en daarna behandeld door de voorzitter van de commissie.

Overwegingen
De METC LDD is van oordeel dat aan alle voorwaarden in artikel 3 van de WMO is voldaan.
De commissie had een aantal vragen over de procedure rond de inclusie in de PRolaCT-O-arm en aansluitend daarop de informatiebrieven voor de verschillende onderzoeksarmen.
Nu de onderzoeker de vragen naar tevredenheid heeft beantwoord en waar nodig de informatiebrieven heeft aangepast, heeft de commissie besloten een positief oordeel af te geven.

Naar de mening van de commissie heeft het amendement geen directe consequenties voor lokale uitvoerbaarheidsaspecten.

De commissie is van oordeel dat het onderzoeksprotocol in een toestemmingsprocedure voorziet die overeenstemt met artikel 6, eerste en derde lid, van de WMO.

De commissie is van oordeel dat is voldaan aan de voorwaarden in artikel 6, vijfde t/m negende lid, van de WMO. De proefpersonen worden op gepaste, volledige en begrijpelijke wijze schriftelijk over het onderzoek geïnformeerd.

Ten slotte wijst de METC LDD u op de verplichtingen die bij het oorspronkelijke positieve besluit zijn vermeld.

Hoogachtend,
Namens de METC Leiden Den Haag Delft,

Mw. P.A. Visser,
secretaris

Leiden, 21 januari 2020

Beroepsprocedure
Tegen dit besluit kan een belanghebbende op grond van artikel 23 van de WMO binnen zes weken na de dag waarop het besluit is bekend gemaakt, administratief beroep instellen bij de Centrale Commissie Mensgebonden Onderzoek (CCMO). Het beroepschrift dient u te adresseren aan CCMO, Postbus 16302, 2500 BH Den Haag.

Bijlage 1

Documenten

A1	Aanbiedingsbrief amendement 2 17-10-2019
A1	Beoordelingsbrief METC amendement d.d. 28-11-2019
A1	Reactie onderzoekers op beoordeling d.d. 09-01-2020
B1	ABR-formulier versie 8 d.d. 17-10-2019
C1	Onderzoeksprotocol amendement 2 versie 3 d.d. 08-10-2019
C2	Amendement 2 d.d. 08-10-2019
E1	Informatiebrief PRolaCT-1 en toestemmingsformulier versie 1.3 d.d. 09-01-2020
E1	Informatiebrief PRolaCT-2 en toestemmingsformulier versie 1.3 d.d. 09-01-2020
E1	Informatiebrief PRolaCT-3 en toestemmingsformulier versie 1.3 d.d. 09-01-2020
E1	Informatiebrief PRolaCT-O en toestemmingsformulier versie 2.0 d.d. 09-01-2020
