# Supplementary material for: The PRolaCT studies — a study protocol for a combined randomised clinical trial and observational cohort study design in prolactinoma
Source: Trials. 2021 Sep 25;22:653. doi: 10.1186/s13063-021-05604-y (PMC8465768; doi:10.1186/s13063-021-05604-y)
Supplement: Supplementary file 5 — Additional file 5. MERC approval amendment PRolaCT-O dated 21 January 2020 (English translation) [file 13063_2021_5604_MOESM5_ESM.pdf]

Our reference: P18.219
CCMO reference: NL63919.058.12
Date: 21st January 2020
Subject: Decision amendment NL63919.058.12

Dear Mrs. Biermasz,

Herewith I send you the decision made by the Medical Research Ethics Committee Leiden The Hague Delft (in Dutch: Medisch-Ethische ToetsingsComissie Leiden Den Haag Delft; METC LDD) regarding the study protocol entitled: **"Prolact – three multicenter prolactinoma randomized clinical trials" (NL639191.058.18)**.

The METC LDD grants her permission for the amendment to named study. For our considerations I refer you to the enclosed decision.

We request you to notify all parties involved with the study about this decision.

We trust that we have sufficiently informed you.

Yours sincerely,
on behalf of the METC Leiden The Hague Delft,

Mrs. P.A. Visser
Secretary

DECREE
Judgement amendment

NL number	NL63919.058.18	CME number	P18.219
Title study	"Prolact – three multicenter prolactinoma randomized clinical trials"		

Contact information: prof. dr. N.R. Biermasz, Endocrinology / AIG, LUMC, Leiden
Sponsor: LUMC, Leiden

Decree
The Medical Research Ethics Committee Leiden The Hague Delft (in Dutch: Medisch-Ethische ToetsingsCommissie Leiden Den Haag Delft; METC LDD) has considered the amendment belonging to abovementioned research file based on article 2, section two, sub a of the Law on Medical scientific research involving humans (in Dutch: Wet Medisch-Wetenschappelijk Onderzoek met mensen; WMO).

The METC LDD has previously approved the study file in the following centers:

- The LUMC in Leiden (Principal Investigator: prof. dr. N.R. Biermasz and dr. W.R. van Furth)
- Amsterdam UMC, location AMC, in Amsterdam (Principal Investigator: dr. J. Hoogmoed)
- Amsterdam UMC, location VUmc, in Amsterdam (Principal Investigator: prof. dr. M.L.
- Drent)
- Elisabeth-Tweesteden Ziekenhuis in Tilburg (Principal Investigator: dr. B. Burhani)
- Radboudumc in Nijmegen (Principal Investigator: dr. H.D. Boogaarts)
- Renier de Graaf Groep (Principal Investigator: dr. C.J. Kapiteijn)

The committee approves the amendment.

Documents
The judgement was based on the documents mentioned in appendix 1.

Background
On 17 October 2019, the amendment has been submitted for approval to the METC LDD. The amendment entails addition of an observational study arm, PRolaCT-O, adjustment of exclusion criteria, adjustment of the recruitment procedure, and adjustment of the registration of *adverse events*. These matters have been approved as of the decision made on 15 November 2019. Subsequently, the patient information letters were adjusted and a patient information letter for PRolaCT-O was added. These information letter have now been judged by the committee.

The amendment has been reviewed by the patient representative and subsequently by the chairman of the committee.

Considerations
The METC LDD judges that all conditions in article 3 of the WMO are met. The committee had some questions regarding the recruitment procedure for PRolaCT-O and the patient information letters for the different study arms. Now the investigator has sufficiently answered the questions, and altered the patient information letters, where needed, the committee reached a positive decision.

According to the committee, the amendment does not hold any consequences regarding local executional aspects.

The committee judges that the research protocol provides a consent procedure that agrees with article 6, sections one and three, of the WMO.

The committee judges that the conditions in article 6, section five to nine, of the WMO, are met. Research subjects are appropriately, completely, and understandably informed in writing about the study, and the possibility to withdraw consent.

Lastly, the CMO points out to you the conditions and obligations named in the initial approval.

Yours sincerely,
on behalf of the Committee Medical Ethics,

Mrs. P.A. Visser
Secretary

APPENDIX 1
Documents

- A1. Cover letter amendment 2 dated 17 Oct 2019
- A1. Assessment letter METC amendment dated 28 Nov 2019
- A1. Answer investigators to assessment dated 09 Jan 2020
- B1. ABR-form version 8 dated 17 Oct 2019
- C1. Study protocol amendment 2 version 3 dated 8 Oct 2019
- C2. Amendment 2 dated 8 Oct 2019
- E1. Subject information letter PRoIaCT-1 and informed consent form version 1.3 dated 9 Jan 2020
- E1. Subject information letter PRoIaCT-2 and informed consent form version 1.3 dated 9 Jan 2020
- E1. Subject information letter PRoIaCT-3 and informed consent form version 1.3 dated 9 Jan 2020
- E1. Subject information letter PRoIaCT-O and informed consent form version 2 dated 9 Jan 2020
