# Supplementary material for: The PRolaCT studies — a study protocol for a combined randomised clinical trial and observational cohort study design in prolactinoma
Source: Trials. 2021 Sep 25;22:653. doi: 10.1186/s13063-021-05604-y (PMC8465768; doi:10.1186/s13063-021-05604-y)
Supplement: Supplementary file 6 — Additional file 6. Grant ZonMw dated 29 June 2017 (original Dutch) [file 13063_2021_5604_MOESM6_ESM.pdf]

ZonMw stimuleert
gezondheidsonderzoek en
zorginnovatie

Laan van Nieuw Oost-Indië 334
2593 CE Den Haag
Postbus 93245
2509 AE Den Haag
Telefoon 070 349 51 11
Fax 070 349 51 00
www.zonmw.nl
info@zonmw.nl

Leids Universitair Medisch Centrum
Divisie 3
Bureau bedrijfsvoering
De heer Drs. E.J. Vos
Locatie J900
Postbus 9600
2300 RC LEIDEN

Dossiernummer
80-84300-98-82021
Ons kenmerk
2017/12881/ZONMW

Datum
29 juni 2017

Contactpersoon
Marleen Jonker
Telefoon 070 349 52 60
doelmatigheidsonderzoek@zonmw.nl

Onderwerp
Honorering van uw subsidieaanvraag, projectnummer 843002806

Geachte heer Vos,

Op 7 maart 2017 heeft u bij ZonMw een subsidieaanvraag ingediend met de titel: *Is endoscopic trans-sphenoidal selective adenoma resection superior and cost-effective compared to medical treatment with cabergoline? A cohort multiple randomized controlled trial for treatment of microprolactinomas*. Met genoegen laat ik u weten dat de commissie Evaluatie van Effecten & Kosten (EEK) van het programma DoelmatigheidsOnderzoek een positief oordeel heeft over uw aanvraag. Dat betekent dat ZonMw u de subsidie zal toekennen. In deze brief leest u hoe ZonMw tot dit oordeel is gekomen en wat u moet doen voordat uw project van start kan gaan.

Beoordeling

Uw aanvraag is beoordeeld op relevantie en op kwaliteit voor de Open Ronde 2018 van het programma DoelmatigheidsOnderzoek, oproep Onderzoek naar reeds toegepaste interventies. Voor deze ronde van het programma ontving ZonMw 19 subsidieaanvragen, waarvan er 7 worden gehonoreerd. De beoordelingsprocedure was als volgt:

Relevantie van de aanvraag voor het programma
De commissie EEK heeft de relevantie van uw subsidieaanvraag voor het programma beoordeeld. Het eindoordeel over de relevantie van uw subsidieaanvraag voor het programma luidt: **relevant**.

Hierbij zijn de volgende opmerkingen van belang:

- De subsidieaanvraag dient te onderbouwen dat de te onderzoeken interventie doelmatig is ten opzichte van de standaard of meest gebruikelijke zorg in Nederland. In de adviesbrief over uw projectidee bent u gevraagd om de doelmatigheidswinst te onderbouwen. De commissie is van mening dat u deze vraag adequaat heeft beantwoord en een realistische schatting is gemaakt van de te behalen doelmatigheidswinst.
- In de oproep is opgenomen dat de studie gericht moet zijn op het genereren van ontbrekende doelmatigheidsgegevens die bruikbaar zijn voor praktijk en/of beleid. De resultaten van deze studie kunnen opgenomen worden in de internationale richtlijn 'Endocrine Society Clinical Guideline for Prolactinomas' en de Nederlandse richtlijn 'Hypofysechirurgie' van de Nederlandse Vereniging voor Neurochirurgie.

- ZonMw hecht veel waarde aan patiëntenparticipatie. Het panel van de Patiëntenfederatie Nederland beoordeelt uw studie als relevant. In uw wederhoor geeft u aan dat u vacatiegeld en reiskosten voor patiëntenparticipatie heeft opgenomen in de begroting van het project.

Kwaliteit van de aanvraag
De commissie EEK heeft ook een eindoordeel over de kwaliteit van uw subsidieaanvraag gegeven. Dit oordeel is gebaseerd op uw aanvraag, de beoordeling hiervan door referenten en uw reactie. Het eindoordeel over de kwaliteit van uw subsidieaanvraag luidt: **voldoende**.

De commissie geeft het volgende argument voor het eindoordeel:
- ZonMw wil geïnvesteerde middelen optimaal laten renderen. Om die reden dient u te overtuigen dat uw project haalbaar is binnen de opgegeven looptijd en budget. In de adviesbrief over uw projectidee bent u gevraagd om de haalbaarheid van de patiënteninclusie te onderbouwen. Ook de referenten vinden dit een belangrijk aandachtspunt. U schat in dat de patiënteninclusie haalbaar is als er objectieve en duidelijke informatie wordt verstrekt aan patiënten. In uw wederhoor geeft u aan dat de deelnemende artsen aan het onderzoek getraind worden voor het counselen van patiënten. Echter, de commissie twijfelt nog steeds of geïnformeerde patiënten zich willen laten randomiseren, aangezien:
  - de bijwerkingen van de medicamenteuze interventie vrij hoog zijn;
  - uit uw online survey blijkt dat 75% van de patiënten, met een prolactinoom, een operatie zou overwegen in plaats van de medicamenteuze interventie.

  De commissie geeft u het voordeel van de twijfel op dit punt, mits er een jaar na de start van de patiënteninclusie een go/no go wordt ingepland.

Op basis van beide eindoordelen heeft de commissie EEK vervolgens een rangschikking van alle aanvragen die voor honorering in aanmerking kwamen gemaakt. Op grond hiervan heeft ZonMw uw aanvraag gehonoreerd.

De programmacommissie verzoekt u bij de uitvoering van het project:
- aandacht te blijven houden voor de communicatie naar en betrokkenheid van patiënten.
- aandacht te hebben voor de haalbaarheid van de patiënteninclusie binnen de looptijd van de studie. U dient ZonMw een jaar na de start van de patiënteninclusie (volgens de planning in uw subsidieaanvraag) te informeren over de stand van zaken van de inclusie. Aan dit moment wordt een beslismoment (go/no go) over de continuering van de studie gekoppeld. Als de patiënteninclusie niet volgens planning verloopt, dan kan dit leiden tot het besluit 'no go' met als gevolg een voorgenomen besluit om de subsidie te beëindigen. Om een helder inzicht te geven in de instroom en doorstroom vragen we u gebruik te maken van de CONSORT-richtlijnen. Bij het voortgangsverslagen vragen we u een CONSORT Flow-diagram toe te voegen; deze krijgt u bij het opvragen van het voortgangsverslag door ZonMw toegestuurd per email.

In het voortgangsverslag en eindverslag dient u op bovenstaande aandachtspunten expliciet in te gaan.

Financiering
Hoogte subsidiebedrag
De financiële bijdrage van ZonMw voor uw project bedraagt maximaal € 454.908,- voor de duur van maximaal 60 maanden. Dit bedrag is inclusief eventueel verschuldigde BTW en exclusief uw cofinanciering van minimaal 10%.

Subsidievoorwaarden
Zoals u weet zijn aan de financiering voorwaarden verbonden. Deze subsidievoorwaarden kunt u downloaden via de website van ZonMw:

www.zonmw.nl/subsidievoorwaarden. Bij patiëntgebonden onderzoek vraagt ZonMw om de gegevens van de studie aan te melden bij het Nederlands Trial Register (NTR). Zie informatie op www.trialregister.nl.

Ik wil u erop wijzen dat ZonMw pas een voorschot uitkeert als aan alle eisen voor het uitvoeren van het onderzoek is voldaan. Ik raad u dan ook aan eventuele procedures hiervoor tijdig te starten. Denkt u bijvoorbeeld aan een positief oordeel van een erkende medisch-ethische toetsingcommissie (METC), de Centrale Commissie Mensgebonden Onderzoek (CCMO), een projectvergunning van de Centrale Commissie Dierproeven (CCD), of een vergunning krachtens de Wet op het Bevolkingsonderzoek (WBO). Als u niet zeker weet of uw project dergelijke verklaringen of vergunningen nodig heeft, kunt u dit nagaan bij de betreffende instanties.

Integriteit
Artikel 2, lid 3 van de Subsidiebepalingen van ZonMw impliceert dat de nationaal en internationaal aanvaarde normen van wetenschappelijk handelen worden nageleefd zoals neergelegd in de Nederlandse Gedragscode Wetenschapsbeoefening (VSNU, laatste herziene versie 31 oktober 2014), dan wel vergelijkbare codes voor niet-universitaire instellingen. In geval van (mogelijke) schending van voornoemde normen bij een door ZonMw gefinancierd project, dient ZonMw hiervan onverwijld op de hoogte te worden gesteld en dienen alle ter zake relevante documenten aan ZonMw te worden overgelegd.

ZonMw bepaalt dat de bijlage Akkoord bekostiging wetenschappelijk onderzoek 2008 en het addendum, conform artikel 7 van het akkoord niet integraal van toepassing zijn op deze subsidie. Deze worden zoveel mogelijk analoog toegepast voor zover het akkoord of het addendum niet strijdig zijn met de Algemene subsidiebepalingen van ZonMw. De Algemene subsidiebepalingen van ZonMw zijn te allen tijde leidend. Zo zal ZonMw bijvoorbeeld altijd afrekenen op basis van werkelijke kosten.

Wat moet u doen?
Belangrijk: schriftelijke bevestiging binnen vier weken
ZonMw kan u een voorschot voor het eerste projectjaar verstrekken. Dit is echter pas mogelijk als u heeft ingestemd met de subsidievoorwaarden en het project daadwerkelijk gestart is. Wilt u daarom vóór 29 juli 2017 schriftelijk onderstaande informatie doorgeven aan ZonMw? Hiervoor kunt u gebruikmaken van het bijgevoegde meldingsformulier:

- uw instemming met de voorwaarden die van toepassing zijn op de toekenning van de financiële bijdrage;
- de startdatum van uw project;
- de bank- en referentiegegevens voor de betalingen van de subsidie;
- Ten aanzien van de goedkeuring van de METC of CCD:
    - Als geen verklaring(en) is vereist, stuurt u een schriftelijke bevestiging hiervan.
    - Als de verklaring(en) noodzakelijk is voor de start van het project, stuurt u de verklaring voor de start van het project aan ZonMw.
    - In het geval de verklaring(en) pas later in het project vereist is, geeft u aan wanneer de verklaring(en) nodig is. Dit is maximaal één jaar na de start van het project. ZonMw keert dan een voorschot uit voor het eerste projectjaar. Verdere voorschotten kan ZonMw alleen betalen als een kopie van de verklaring(en) is ontvangen.

Ik wijs u erop dat het project **uiterlijk zes maanden** na dagtekening van deze brief moet beginnen. Gaat het project later van start, dan vervalt de honorering van uw aanvraag. Hiervan kan alleen in zeer bijzondere gevallen worden afgeweken.

Publiekssamenvatting
ZonMw publiceert alle gehonoreerde projecten op haar website met een leesbare Nederlandse samenvatting. Deze is bedoeld voor een breed geïnteresseerd publiek met verschillende achtergronden, op taalniveau eind VWO. Zie de schrijfwijzer op http://www.zonmw.nl/nl/over-zonmw/logo-huisstijl.
Wij verzoeken u deze Nederlandse publiekssamenvatting zo spoedig mogelijk, maar tenminste binnen vier weken na dagtekening van deze brief aan te leveren. Hiervoor kunt u in ProjectNet het tekstvak Publiekssamenvatting gebruiken (maximaal 150 woorden / 1.000 karakters inclusief spaties).

Voortgangsverslag
ZonMw wil graag op de hoogte blijven van de voortgang van uw project. Van het programmasecretariaat ontvangt u 18 maanden na startdatum het verzoek een voortgangsrapportage in te dienen. ZonMw werkt met een verkorte voortgangsrapportage die u halverwege het project indient (tenzij anders bepaald). Daarnaast bent u verplicht tussentijdse wijzigingen te melden aan ZonMw. Pas na goedkeuring door ZonMw zijn de wijzigingen toegestaan.

Kennisbenutting
Resultaten van het project kunnen toepassing vinden in de praktijk, maar ook een rol spelen bij het maken van beleid, een volgende stap vormen in een wetenschappelijke carrière of de basis vormen voor een nieuw project. Om aan te geven wat er met de resultaten gebeurt, stellen wij u in voortgangs- en eindverslag diverse vragen over verspreiding- en implementatie. Ook dienen publicaties over en resultaten van het project tot vier jaar na afronding via ProjectNet aan ZonMw te worden aangeboden. Daarnaast bent u verplicht om ZonMw in deze periode te informeren over het gebruik van de resultaten.

Datamanagement
Op basis van artikel 20 van de Subsidiebepalingen van ZonMw moeten alle subsidieontvangers een datamanagementplan opstellen. Wij ontvangen graag uiterlijk 29 september 2017 een eerste versie. Het format voor het datamanagementplan en de uitleg erbij is te vinden op www.zonmw.nl/ttd. U kunt het als PDF toesturen naar doelmatigheidsonderzoek@zonmw.nl. Gedurende uw project kunt u wijzigingen of aanvullingen doorvoeren. Indien u geen dataverzameling opbouwt, kunt u dat melden bij het programmateam.

Mocht u nog vragen hebben over deze brief, neemt u dan gerust contact op met de medewerker die in het briefhoofd vermeld staat. Als u ontevreden bent over de wijze waarop ZonMw uw aanvraag heeft behandeld, kunt u een klacht indienen (zie hieronder). Vermeld in uw communicatie met ZonMw altijd het projectnummer. Nu uw aanvraag gehonoreerd is, vervalt het oorspronkelijke nummer en geldt het nieuwe projectnummer: 843002806.

Ik wil u nogmaals feliciteren met de honorering van uw subsidieaanvraag. Veel succes bij de uitvoering van uw project!

Met vriendelijke groet,
namens het bestuur,

Henk J. Smid
directeur bw

Ons kenmerk
2017/12881/ZONMW
Datum
29 juni 2017
Dossiernummer
80-84300-98-82021

Bijlage(n)
Meldingsformulier start project

Kopie
Dr. W.R. van Furth, Leids Universitair Medisch Centrum

Tegen deze beschikking kunt u bezwaar maken. In dat geval stuurt u binnen zes weken na de dag waarop het besluit bekend is gemaakt een bezwaarschrift aan het bestuur van ZonMw, t.a.v. Commissie Bezwaarschriften ZonMw, Postbus 93 245, 2509 AE Den Haag. Meer informatie over signaleren, klagen en bezwaar maken? Raadpleeg de website: www.zonmw.nl/signalerenklagenbezwaarmaken
