# Supplementary material for: The PRolaCT studies — a study protocol for a combined randomised clinical trial and observational cohort study design in prolactinoma
Source: Trials. 2021 Sep 25;22:653. doi: 10.1186/s13063-021-05604-y (PMC8465768; doi:10.1186/s13063-021-05604-y)
Supplement: Supplementary file 7 — Additional file 7. Grant ZonMw dated 29 June 2017 (English translation) [file 13063_2021_5604_MOESM7_ESM.pdf]

File number 80-84300-98-82021
Date 29th June 2017

Subject
Honorarium of grant proposal, project number 843002806

Dear mister Vos,

On March 7th 2017 you have submitted a grant proposal titled *'Is endoscopic transsphenoidal selective adenoma resection superior and cost-effective compared to medical treatment with cabergoline? A cohort multiple randomized controlled trial for treatment of microprolactinomas.'* to ZonMw. I am pleased to inform you that the committee 'Evaluation of Effects & Costs (EEK)' of the 'DoelmatigheidsOnderzoek' program has positively judged your submission. This means that ZonMw will honor you a grant. In this letter you can read how ZonMw has come to this decision and what is required before your project can start.

Evaluation
Your proposal has been evaluated on relevance and quality for the 'Open Round 2018' of the 'DoelmatigheidsOnderzoek' program, call for studies investigating existing interventions. For this round, ZonMw has received 19 proposals, of which 7 will be granted. The evaluation process was as follows:

Relevance of your proposal for the program
The EEK committee has evaluated the relevance of your proposed study for the grant program. The final evaluation of your proposal for the program is: **relevant**.
The following comments are of importance:

- The grant proposal should deliver supporting evidence that the intervention is more effective in comparison with the standard or usual care in the Netherlands. In the advisory letter regarding your project idea, you have been askes to support the added effectiveness. The commission is of opinion that you have sufficiently answered this question and have made a realistic estimation of the added effectiveness.
- The call for proposals required studies to generate missing effectiveness data that can be used in daily practice and/or policy development. The results of this study can be incorporated in the international guideline *'Endocrine Society Clinical Guideline for Prolactinomas'* and the Dutch guideline *'Hypofysechirurgie'* (Pituitary surgery) of the Dutch Association for Neurosurgery.
- Patient participation is of great value to ZonMw. The panel of the Patient federation Netherlands deems your study as relevant. In your rebuttal you state that you have not included costs for vacation days and travel costs for patient participation in the budget for the project.

Quality of the proposal
The EEK committee has also given a final evaluation on the quality of your grant proposal. This evaluation is based on your proposal, review by referents, and your rebuttal. The final evaluation of the quality of your grant proposal is deemed: **adequate**.
The committee gives the following argumentation for this evaluation:

- ZonMw aims at optimal rendering of investments. Therefore, you are asked to argument that your project is feasible within the given time and with the given budget. In the advisory letter regarding your project proposal, you have been asked to argument the feasibility of patient

inclusion. The referents reviewing your proposal also think this is an importance matter of attention. You estimate that patient inclusion is feasible if patients receive objective and clear information. In your rebuttal, you state that doctors participating with this study will be trained to counsel patients for study inclusion. Nonetheless, the committee still doubts if informed patients will be willing to randomize, since:

- side effects of the medical treatment are quite high;
- your online survey shows that 75% of prolactinoma patients would consider surgery instead of medical treatment.

The committee will give you the benefit of the doubt regarding this point, under the condition that a go/no go point is planned one year after start of patient inclusion.

The EEK committee has ranked all qualified proposals based on the final evaluations. Based on this ranking, ZonMw has honored your grant proposal.

The committee requests that during the execution of this project, you:

- Maintain attention for communication with and involvement of patients.
- Have attention for the feasibility of patient inclusion within the duration of the study. You are required to inform ZonMw about your progress one year after the start of patient inclusion (according to the planning in your grant proposal). A decisive moment (go/no go) about continuation of the study is linked to this moment. If patient inclusion is not running according to planning, this may lead to a 'no go' decision and subsequently an intention to terminate the grant. In order to give a clear insight in patient recruitment, we ask you to use the CONSORT guidelines. We ask you to include a CONSORT flow diagram with progress reports, you will receive these by email from ZonMw when progress reports are requested.

You are required to explicitly address these points of interest in your progress reports.

Financing

Grant budget

The financial contribution for your project from ZonMw is maximum € 454,908.00 for a duration of maximum 60 months. This amount includes any applicable taxes and is excluding your co-financing of at least 10%.

Grant conditions

As you know, this financing is subject to conditions. You can download these grant conditions from the ZonMw website: www.zonmw.nl/subsidievoorwaarden. In case of studies with patients, ZonMw asks to register the study in the Dutch Trial Registry (NTR). See www.trialregister.nl for information.

I want to point out that ZonMw will not make a deposit until all regulatory demands for the study are met. I therefore recommend a timely start of any applicable procedures. For example, think of a positive judgement from an official medical ethical committee (METC), the central committee for studies involving humans (CCMO), a project license from the central committee animal studies (CCD), or a license under the law involving public health studies. If you are not sure if your project requires such declarations or licenses, you can check this with the specific authorities.

Integrity

Artikel 2, section 3 of the grant conditions of ZonMw implies that national and international applicable standards of scientific conduct are met as addressed in the Dutch Code of scientific conduct (VSNU, most recent update 31 October 2014), or comparable codes for institutions not linked to a university. In case of (potential) violation of aforementioned standards in a by ZonMw

financed project, ZonMw should be informed without delay, and all relevant documents should be handed to ZonMw.

ZonMw decides that the appendix Agreement of financing scientific research 2008 and the addendum, conform article 7 of this agreement are not integrally applicable to this grant. These are applied analogous as far as the Agreement or addendum are not inconsistent with the General grant conditions of ZonMw. The General grant conditions of ZonMw are leading at all times. For instance, ZonMw will always payout based on true costs.

What do you have to do?
Important: written confirmation within four weeks
ZonMw can provide a deposit for the first year. This is, however, possible after you have agreed with the grant conditions and the project has started. Will you thus provide us with the information listed below before 29 July 2017? For this, you may use the enclosed form:

- your agreement to the conditions applicable to granting of the financial contribution;
- the start date of your project;
- bank account and reference data for payment of the grant;
- regarding METC or CCD approval:
    - In case no declaration(s) is required, you have to send a written confirmation of this.
    - In case the declaration(s) is required for the start of your project, you have to send the declaration to ZonMw before the start of your project.
    - In case the declaration(s) is required later on during the project, you have to state when the declaration(s) is required. This is maximum one year after the start of the project. ZonMw will provide a deposit for the first year of the project. Further deposits can only be paid by ZonMw when a copy of the declaration(s) has been received.

I point out to you that the project has to start **within six months** after date of this letter. If the project starts later, the honorarium of your proposal will expire. Only in highly extraordinary cases will be deviated from this.

Public summary
ZonMw publishes all granted projects on her website with an easily readable Dutch summary. This is intended for a broad interested public with varying backgrounds, with a top level high school (VWO) language level. See the writing instruction on http://www.zonmw.nl/nl/over-zonmw/logo-huisstijl.
We request that you provide this Dutch public summary as soon as possible, but at least withinfour weeks after date of this letter. For this you can use the textbox Public summary in ProjectNet (maximum 150 words or 1000 characters including spaces).

Progress reports
ZonMw wants to stay up-to-date about the progress of your project. 18 months after the start of your project, you will receive a request to provide a progress report from the program secretary staff. ZonMw works with a short progress report that you provide halfway through the project (unless decided differently). You are furthermore required to inform ZonMw of any changes to the project. Changes are allowed only after approval by ZonMw.

Use of knowledge
Results of the project can be applicable in daily practice, but may also playa part in policy development, form a next step in a scientific career, or form the base of a new project. To state what is done with the results, we ask you several questions regarding distribution and implementation in progress- and final reports. Publications of results of the project should furthermore be handed to

ZonMw via ProjectNet until four years after project completion. You are also required to inform ZonMw in this period about use of the results.

Data management
Based on article 20 of the grant conditions of ZonMw, should all grant recipients make a data management plan. We would like to receive a first draft before 29 September 2017. The format for the data management plan and the accompanying explanation can be found on www.zonmw.nl/ttd. You can send it in PDF format to doelmatigheidsonderzoek@zonmw.nl. Changes or additions can be made during the course of the project. If you will not build a data collection, u can inform the program team.

In case you have any questions about this letter, you are welcome to contact the employee named in the letter head. If you are unsatisfied about the way ZonMw has addressed your proposal, you can file a complaint (see below). Always state your project number in your communication with ZonMw. Now your grant proposal has been honored, the original number will expire and a new project number is effective: 843002806.

I want to again congratulate you with the honorarium of your grant proposal. Good luck with the execution of your project!

Yours sincerely,
on behalf of the board,

Henk J. Smid
directeur
